# Constructing a Superlithiophilic 3D Burr‐Microsphere Interface on Garnet for High‐Rate and Ultra‐Stable Solid‐State Li Batteries

**DOI:** 10.1002/advs.202207056

**Published:** 2023-02-15

**Authors:** Butian Chen, Jicheng Zhang, Tianran Zhang, Ruoyu Wang, Jian Zheng, Yanwu Zhai, Xiangfeng Liu

**Affiliations:** ^1^ Center of Materials Science and Optoelectronics Engineering College of Materials Science and Optoelectronic Technology University of Chinese Academy of Sciences Beijing 100049 P. R. China; ^2^ CAS Center for Excellence in Topological Quantum Computation University of Chinese Academy of Sciences Beijing 100190 P. R. China

**Keywords:** 3D burr‐microsphere, garnet, interface modification, solid‐state batteries

## Abstract

Garnet‐type solid‐state electrolyte (SSE) Li_6.5_La_3_Zr_1.5_Ta_0.5_O_12_ attracts great interest due to its high ion conductivity and wide electrochemical window. But the huge interfacial resistance, Li dendrite growth, and low critical current density (CCD) block the practical applications. Herein, a superlithiophilic 3D burr‐microsphere (BM) interface layer composed of ionic conductor LiF‐LaF_3_ is constructed in situ to achieve a high‐rate and ultra‐stable solid‐state lithium metal battery. The 3D‐BM interface layer with a large specific surface area shows a superlithiophilicity and its contact angle with molten Li is only 7° enabling the facile infiltration of molten Li. The assembled symmetrical cell reaches one of the highest CCD (2.7 mA cm^−2^) at room temperature, an ultra‐low interface impedance of 3 Ω cm^2^, and a super‐long cycling stability of 12 000 h at 0.1–1.5 mA cm^−2^ without Li dendrite growth. The solid‐state full cells with 3D‐BM interface show outstanding cycling stability (LiFePO_4_: 85.4%@900 cycles@1 C; LiNi_0.8_Co_0.1_Mn_0.1_O_2_:89%@200 cycles@0.5 C) and a high rate capacity (LiFePO_4_:135.5mAh g^−1^ at 2 C). Moreover, the designed 3D‐BM interface is quite stable after 90 days of storage in the air. This study offers a facile strategy to address the critical interface issues and accelerate the practical application of garnet‐type SSE in high performance solid‐state lithium metal batteries.

## Graphical abstract

A superlithiophilic 3D burr‐microsphere interface on garnet for high‐rate and ultra‐stable solid‐state Li batteries is constructed. The symmetric battery shows a high critical current density of 2.7 mA cm^−2^, an ultra‐low interface impedance of only 3 Ω cm^2^, and a super‐long cycling stability of over 12 000 h. The full cells show an outstanding cycling stability and high rate capacity.

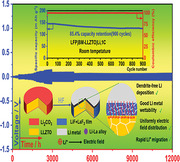

## 1 Introduction

With the rapid development and application of electric vehicles, the demand for batteries with high energy density and high safety becomes more and more urgent.^[^ ^1^, ^2^, ^3^, ^4^ ^]^ Nevertheless, the traditional liquid electrolyte‐based lithium‐ion battery cannot meet the requirements of future electric vehicles.^[^ ^5^, ^6^, ^7^, ^8^ ^]^ Solid‐state batteries (SSBs) using solid‐state electrolytes (SSEs) have attracted great attention because of their high energy density and high safety.^[^ ^9^ ^]^ SSE is the core component of a solid‐state battery. Among the different types of solid electrolytes (sulfides,^[^ ^10^, ^11^, ^12^ ^]^ polymer/inorganic composites,^[^ ^13^, ^14^, ^15^, ^16^ ^]^ oxides,^[^ ^17^, ^18^, ^19^ ^]^ and halides^[^ ^20^, ^21^, ^22^ ^]^), oxide solid electrolytes especially garnet‐type Li_7_La_3_Zr_2_O_12_ (LLZO, when Ta is doped, it is LLZTO—Li_6.5_La_3_Zr_1.5_Ta_0.5_O_12_) has attracted much interest owing to the high ionic conductivity, high electrochemical window, and high thermal stability.^[^ ^18^, ^19^ ^]^ But the solid–solid interface incompatibility, the huge interfacial resistance, and Li dendrite growth on Li|LLZTO interfaces critically blocked the practical applications. Some groups reported that the Li_2_CO_3_ formed on the surface of LLZTO resulted in poor interface incompatibility and huge interfacial resistance.^[^ ^23^, ^24^, ^25^, ^26^, ^27^, ^28^, ^29^, ^30^ ^]^ Some strategies have been extensively explored to remove Li_2_CO_3_ and improve interface incompatibility.^[^ ^30^, ^31^, ^32^ ^]^ For example, Zhang et al. transformed Li_2_CO_3_ on the surface of LLZTO powder into LiCoO_2_ (LCO) by a Li donor reaction,^[^ ^30^ ^]^ which facilitates Li^+^ transport on the interface. But the Li dendrite growth of LLZO‐based solid‐state batteries during a long‐term cycle is still a safety issue.^[^ ^33^, ^34^, ^35^ ^]^ The reason for the Li dendrite growth is not quite clear but the incomplete contact between the electrode and electrolyte, and the resultant uneven current distribution are considered to be largely responsible for the dendrite growth.^[^ ^36^ ^]^ Krauskopf et al. reported that the high nucleation and inhomogeneous Li deposition led to dendrite growth and short circuits.^[^ ^37^ ^]^ Li et al.^[^ ^38^ ^]^ consider that the overpotential distribution on the Li|LLZTO interface is responsible for the inhomogeneous Li deposition and Li dendrites.

Various strategies have been applied to improve the interface compatibility between Li metal and LLZO. For example, SnN*_x_*,^[^ ^39^ ^]^ Al_2_O_3_,^[^ ^40^ ^]^ Ge,^[^ ^41^ ^]^ Sb,^[^ ^42^ ^]^ Cu_3_N,^[^ ^43^ ^]^ graphite,^[^ ^44^ ^]^ Ga,^[^ ^45^ ^]^ Li_2_PO_2_F_2_,^[^ ^46^ ^]^ NH_4_H_2_PO_4_,^[^ ^47^ ^]^ and Li_3_PO_4_^[^ ^48^ ^]^ have been used to reduce the interface resistance between LLZO and Li metal anode. In addition, the surface energy of Li metal can be changed by alloying to get a better wettability to LLZO. For instance, Li—Na,^[^ ^49^ ^]^ Li—C,^[^ ^50^ ^]^ Li—C_3_N_4_,^[^ ^51^ ^]^ Li‐BN,^[^ ^52^ ^]^ and Li—Si_3_N_4_^[^ ^53^ ^]^ have improved the wettability of Li metal. But the critical current density (CCD) of the solid‐state batteries in most of the studies is usually less than 1.0 mA cm^−2^, which limits the high‐rate performance of SSBs.^[^ ^54^ ^]^ Just recently, Yang et al. reduced the interface resistance to 5.1 Ω cm^2^ through Li_2_PO_2_F_2_ treatment and the CCD was increased to 1.2 mA cm^−2^. The lithium symmetric battery was stably cycled for 1500 h at a current density of 0.6 mA cm^−2^.^[^ ^46^ ^]^ Almost at the same time, Guo et al. modified the interface via NH_4_H_2_PO_4_ to reduce the interface resistance to 13 Ω cm^2^ and a CCD of 1.2 mA cm^−2^ was also achieved. The lithium symmetric battery could stably run 1000 h at 0.1 mA cm^−2^.^[^ ^47^ ^]^ How to further enhance the CCD and the high‐rate capability of SSBs as well as improve the long‐term cycling stability is still a big challenge for garnet‐type SSEs. In addition, the storage instability of garnet‐type SSEs in the air is also a critical issue in practical applications.

**Scheme 1:**
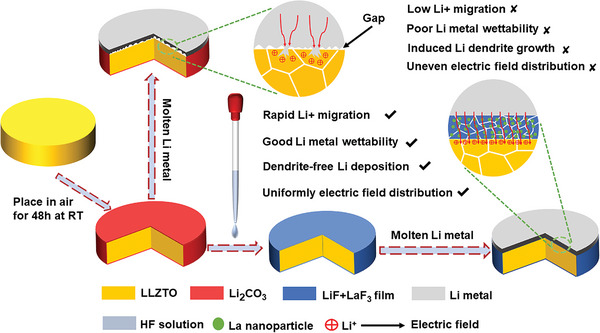
The schematic of the constructing process of and protected Li|LLZTO interface for Li metal solid batteries.

Herein, we design a superlithiophilic 3D burr‐microsphere (3D‐BM) interface layer composed of ionic conductor LiF—LaF_3_ on the Li|LLZTO interface via a facile HF treatment (defined as BM‐LLZTO) (**Scheme** **1**), which simultaneously addresses the critical issues of low CCD, inferior rate performance and insufficient long‐term cycling instability. The storage stability is also significantly improved after the construction of 3D‐BM. The formed 3D‐BM structure has a very large specific surface area, which is quite favorable for the infiltration of molten Li, and its contact angle with molten Li is only 7°, showing a superlithiophilicity. Furthermore, part of the ionic conductor LaF_3_ in the 3D‐BM interface layer reacts with molten Li to form electron conductor metal La and Li—La alloy (When BM‐LLZTO reacts with molten Li, it is marked as Li‐BM‐LLZTO), and transformed into a mixed ion‐electronic conductor (MIEC). This lowers the nucleation/diffusion energy barrier, uniforms the electric field distribution, and decreases the local current density. The assembled symmetrical cell reaches a record CCD of 2.7 mA cm^−2^, an ultra‐low interface impedance (only 3 Ω cm^2^), and super‐long cycling stability (over 12 000 h at 0.1–1.5 mA cm^−2^) without Li dendrite growth at room temperature. To the best of our knowledge, 12 000 h is the reported longest cycle time for SSBs at room temperature. The discharge capacity of LiFePO_4_‐based solid‐state full battery at 0.1 C and 2 C is 159.5 and 135.5 mAh g^−1^, respectively. The capacity retention rate after 900 cycles at 1 C is 85.4% and the Coulombic efficiency is as high as 99.9%. Similarly, the discharge capacity of LiNi_0.8_Co_0.1_Mn_0.1_O_2_‐based solid‐state full battery at 0.1 C and 2 C is 203.5 and 114.8 mAh g^−1^, respectively, and the capacity retention and Coulombic efficiency of 200 cycles at 0.5 C are 89% and 98.6% respectively. Moreover, the designed interface structure is quite stable in air and no Li_2_CO_3_ is regenerated on the surface of LLZTO after 90 days of storage. This work offers a facile and effective way to simultaneously solve the Li|LLZTO interface issues for high‐performance solid‐state Li metal batteries, which greatly promotes the practical application of solid‐state batteries.

## 2 Results and Discussion

### 2.1 Characterization of LLZTO Pellets

The LLZTO pellets are prepared by the traditional high‐temperature solid‐phase method and sintered in the air without pressure. The phase structure obtained is verified by X‐ray diffraction (XRD), so the diffraction peak matches PDF#45‐0109 (Figure S1, Supporting Information), and no other impurity peaks appear. Figure S2, Supporting Information, is a scanning electron microscope (SEM) image of a section of LLZTO, it can be seen from Figure S2, Supporting Information, that the average crystal grain size of LLZTO is about 3 µm, and the crystal grains are in very close contact, and no obvious pores are observed, which proves that the prepared LLZTO ceramic pellets are very dense (over 98% measured by the Archimedes method). The electrical conductivity at room temperature is 8.4 × 10^−4^ S cm^−1^ by electrochemical impedance spectroscopy (EIS) (Figure S3, Supporting Information). The EIS is tested at 25–100 °C, and the corresponding activation energy, *E*_a_ is calculated to be 0.37 eV (Figure S4, Supporting Information). To explore the consistency and repeatability of LLZTO ceramic pellets in the sintering process, 40 ceramic pellets were sintered at one time and their conductivity was tested. As shown in Figure S5, Supporting Information, the conductivity of 40 ceramic pellets ranged from 7.5 to 8.9 × 10^−4^ S cm^−1^, and the mode was 8.4 × 10^−4^ S cm^−1^. The above results show that the prepared LLZTO ceramic pellets are relatively successful, with high consistency and repeatability, which are very important for subsequent experiments.

### 2.2 The Mechanism of 3D‐BM (BM‐LLZTO) Structure to Stabilize Li|LLZT Interface

**Figure 1:**
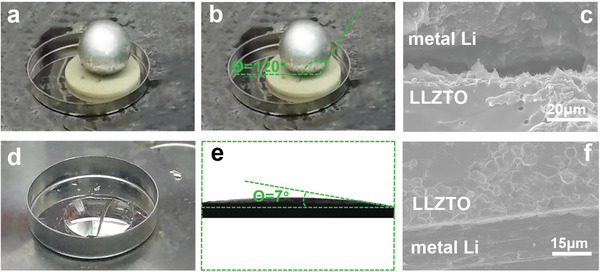
a–c) Optical spectrum, contact angle measurement, and SEM image of LLZTO and molten lithium infiltration. d–f) Optical spectrum, contact angle measurement, and SEM image of BM‐LLZTO and molten Li infiltration.

In general, the contact between solid Li and LLZTO is difficult to achieve, hence the Li**|**LLZT interface was constructed by the molten Li method.^[^ ^55^ ^]^ The wettability of LLZTO and BM‐LLZTO to molten Li was tested at 240 °C, respectively. Due to the very poor wettability, the molten Li shrinks into a small ball on the LLZTO surface (**Figure** **1**a), and its contact angle is about 120° (Figure 1b), which means the lithiophobic property of the LLZTO surface. Meanwhile, very large voids can be observed at the Li**|**LLZT interface (Figure 1c), which further verifies the lithiophobicity of the LLZTO surface. On the contrary, BM‐LLZTO obtained by HF treatment at different time showed good lipophilicity, and the minimum contact angle measured after 15 min treatment was only 7°, showing very good wettability and superlipophilicity (Figure 1d,e and Figure S6, Supporting Information). The reasons for the good wettability will be discussed later. It can be seen from the SEM image of the interface that BM‐LLZTO is in close contact with lithium metal without any voids (Figure 1f). Meanwhile, after 15 min of HF treatment, the Li^+^ conductivity decreased, but still reached a high Li^+^ conductivity of 4.7 × 10^−4^ S cm^−1^ (Figure S7, Supporting Information).

**Figure 2:**
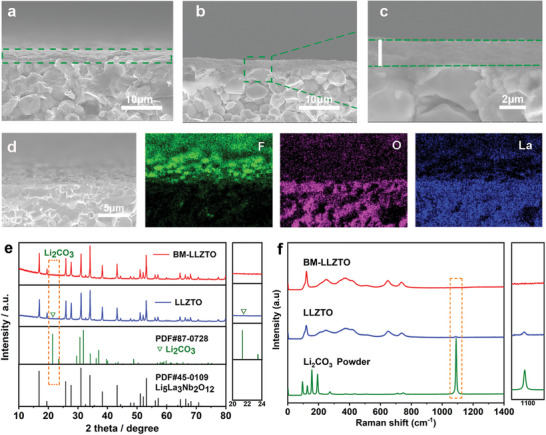
a–c) SEM of LLZTO (a) and BM‐LLZTO (b,c) cross‐sections. d) SEM‐EDX of BM‐LLZO pellet cross‐sections. e) XRD patterns of LLZTO and BM‐LLZTO after being placed in the air for 48 h. f) Raman spectra of LLZTO and BM‐LLZTO after being placed in the air for 48 h.

SEM is further applied to observe the cross‐sectional morphology of LLZTO and BM‐LLZTO. In the LLZTO section, it can be seen that the internal ceramic grains are arranged very tightly, and there are no obvious voids, showing a very high density, which is consistent with the result shown in Figure S2, Supporting Information. However, a thick layer of Li_2_CO_3_ is formed on the surface of LLZTO due to being placed in the air for 48 h (**Figure** **2**a). For a better comparison, LLZTO was placed in the air for 48 h and then treated with a 40% HF solution to obtain BM‐LLZTO. Through the energy dispersive spectrometer (EDS) test on the section of BM‐LLZTO, the F element in BM‐LLZTO is mainly distributed on the surface, indicating that a thick fluoride layer is formed on the surface of BM‐LLZTO, and the thickness is about 2 µm (Figure 2b,c). The La and O elements that represent LLZTO mainly exist inside BM‐LLZTO, and no strong signals of La and O elements are detected on the surface. The reason is that the fluoride layer on the surface blocks its signals (Figure 2d). To further characterize the structural characteristics of LLZTO and BM‐LLZTO, XRD was used to test the surface of LLZTO and BM‐LLZTO. As shown in Figure 2e, the main phases of LLZTO and BM‐LLZTO are cubic garnet phases (PDF#45‐0109), but a clear Li_2_CO_3_ diffraction peak (PDF#87‐0728) at 21.3° appears in the sample of LLZTO. In BM‐LLZTO, the diffraction peak of Li_2_CO_3_ is not observed, all the diffraction peaks belong to LLZTO, and there is no miscellaneous peak. It can also be confirmed by the Raman spectra of LLZTO and BM‐LLZTO (Figure 2f). The peaks at 243, 375, 645, and 728 cm^−1^ were characteristic peaks of the cubic garnet phase, and the first two peaks were related to the Li—O bonding in the garnet structure.^[^ ^52^ ^]^ The peak of LLZTO at 1087 cm^−1^ comes from Li_2_CO_3_ produced on its surface. It is proved that LLZTO can generate a large amount of Li_2_CO_3_ on the surface when placed in air for 48 h, and the results are consistent with those shown in Figure 2a. On the contrary, no peak of Li_2_CO_3_ was observed on the BM‐LLZTO surface because Li_2_CO_3_ on the BM‐LLZTO surface was completely removed.

To explore whether BM‐LLZTO can inhibit the formation of Li_2_CO_3_, fresh LLZTO and BM‐LLZTO were placed in the air for 90 days, and XRD and Raman characterization were carried out before and after placement. As Figure S8, Supporting Information, shows, after being placed for 90 days, LLZTO showed strong Li_2_CO_3_ diffraction peaks at 21.3°, 23.5°, and 37°, while the diffraction peaks of BM‐LLZTO were the same as 90 days ago, and there was no Li_2_CO_3_ impurity peak. The same result is also shown in the Raman spectrum, no Li_2_CO_3_ peak was observed after BM‐LLZTO was placed in the air for 90 days, while obvious Li_2_CO_3_ peaks appeared at 158 and 1087 cm^−1^ after LLZTO was placed for 90 days (Figure S9, Supporting Information). The above results indicate that BM‐LLZTO can be stored in air for a long time without generating Li_2_CO_3_, which greatly improves the storage stability of LLZTO in the air, and effectively solves the storage problem of LLZTO solid electrolytes in practical applications.

**Figure 3:**
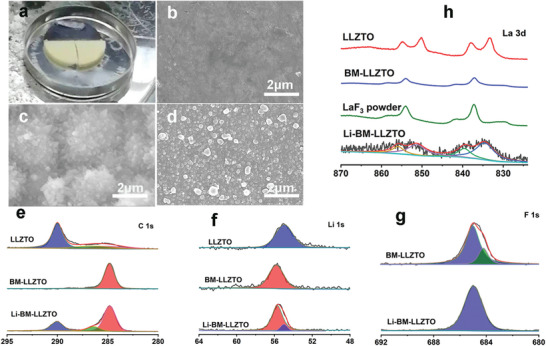
a) Fresh LLZTO pellet is divided into three parts optical image. Surface SEM images of b) LLZTO, c) BM‐LLZTO, and d) Li‐BM‐LLZTO. e–g) XPS map of the surface of LLZTO, BM‐LLZTO, and Li‐BM‐LLZTO.

To explore the formation mechanism of BM‐LLZTO and the reason why the contact between BM‐LLZTO and Li metal produces a good interface, BM‐LLZTO reacts with molten Li at 240 °C to obtain Li‐BM‐LLZTO. To better study the effect of HF solution on LLZTO, the fresh LLZTO pellets are divided into three parts: the upper part is Li‐BM‐LLZTO, the lower‐left part is LLZO, and the lower right part is BM‐LLZTO (**Figure** **3**a). On the surface of Li‐BM‐LLZTO, metallic Li will diffuse along the surface of BM‐LLZTO, and spread to the entire surface with the extension of heating time, and the surface will change from light yellow to navy blue (Figure 3a). The reason why the surface of Li‐BM‐LLZTO appears navy blue may be due to the formation of metallic La on the surface, and it is speculated that the reaction process is as follows:

(1) $${\mathrm{Li}}_{2}{\mathrm{CO}}_{3}+2\mathrm{HF}\to 2\mathrm{LiF}+H_{2}O+CO_{2}$$

(2) $$\begin{array}{ccc} & & {\mathrm{Li}}_{6.5}{\mathrm{La}}_{3}{\mathrm{Zr}}_{1.5}{\mathrm{Ta}}_{0.5}O_{12}+2\left(x+y\right)\mathrm{HF}\overset{x\geq y}{\underset{x\leq 6.5,\;y\leq 3}{\to }}\\ & & {\mathrm{Li}}_{6.5-x}{\mathrm{La}}_{3-y}{\mathrm{Zr}}_{1.5}{\mathrm{Ta}}_{0.5}O_{12-x-y}F_{x-y}+x\mathrm{LiF}+y{\mathrm{LaF}}_{3}+\left(x+y\right)H_{2}O \end{array}$$

(3) $${\mathrm{LaF}}_{3}+3\mathrm{Li}\to 3\mathrm{LiF}+\mathrm{La}$$

To prove the feasibility of the above three chemical equations, we calculated the Gibbs free energy of the chemical reaction equation. The standard Gibbs free energy of chemical reaction Equation (1) is −127.414 KJ mol^−1^ (Table S1, Supporting Information), which means that the reaction is a spontaneous process. For Equation (3), when the temperature is in the range of 298.15–600 K, the calculated Δ*r G*_m_ < 0 (Table S1, Supporting Information), proves that the reactions are spontaneous in this range. The spontaneous diffusion mechanism can ensure the uniform distribution of Li^+^ at the interface, to make the current distribution uniform during the cycle, which has a positive effect on the inhibition of lithium dendrites. The Gibbs free energy of chemical reaction Equation (2) is calculated by density functional theory (DFT). The results show that the Gibbs free energy distributions are −20.02 KJ mol^−1^ (Table S2, Supporting Information) and −45.94 KJ mol^−1^ (Table S3, Supporting Information) when (*x* = 3.25, *y* = 1.5) and (*x* = 6.5, *y* = 3), which proves that under restricted conditions, no matter what the values of *x* and *y* are, the reaction can proceed spontaneously on thermodynamics.

On the other hand, A large number of 3D‐BM structures can be seen on the surface of BM‐LLZTO (Figure 3c and Figure S10a–c, Supporting Information). The reason for its formation is that after removing the Li_2_CO_3_ on the surface, the high‐concentration HF solution continues to corrode the LLZTO pellets along the grain boundaries and remove impurities from grain boundaries,^[^ ^41^ ^]^ forming a 3D‐BM structure on the surface. The EDS characterization (Figure S10d, Supporting Information) results show that the 3D‐BM is composed of fluoride, and its structure is consistent with the cross‐sectional characterization (Figure 2d). On the surface of Li‐BM‐LLZTO, a large number of small spherical particles are formed (Figure 3d). These small balls are composed of LiF and metal La formed by the reaction of the metal Li and LaF_3_. To further determine the specific components of the reaction process, X‐ray photoelectron spectroscopy (XPS) was used for characterization. As shown in Figure 3e, in the C 1s spectra, two peaks at binding energies of 284.8 and 290 eV were detected for the LLZTO pellet, which can be assigned to adventitious carbon and Li_2_CO_3_, respectively. There is no Li_2_CO_3_ peak on the BM‐LLZTO surface, which means that Li_2_CO_3_ on the surface has been completely removed. On the surface of Li‐BM‐LLZTO, a Li_2_CO_3_ peak was observed again, the reason is that the XPS sample was prepared in air and there is incompletely reacted metal Li on the surface of Li‐BM‐LLZTO, which reacts with CO_2_ in the air to form Li_2_CO_3_ again. In the Li 1s spectra (Figure 3f), the lower binding energy of 54.5 eV can be assigned to the Li—O bond in LLZTO, and the binding energy of 55.7 eV belongs to LiF. In addition to the binding energy of 55.0 eV in the Li 1s spectra, the binding energy of Li‐BM‐LLZTO at 55.0 eV belongs to Li_2_CO_3_, which corresponds to the binding energy of 290 eV in the C 1s spectrum. Figure 3g is the F 1s spectrum, the binding energy at 685.0 eV comes from LiF, while in BM‐LLZTO, except LiF, the binding energy at 684.2 eV comes from LaF_3_.^[^ ^56^ ^]^ In La 3d spectrum (Figure 3h), the La^3+^ 3d_3/2_ and 3d_5/2_ region in LLZTO have four subpeaks at 833.3, 837.2, 850.1, and 854.0 eV because of the spin‐orbit multiplet splitting.^[^ ^57^ ^]^ The La 3d spectrum in BM‐LLZTO is the same as that in LaF_3_ powder, which means that La 3d on the surface of BM‐LLZTO comes from LaF_3_, and the result is consistent with the binding energy at 684.2 eV in the F 1s spectrum (Figure 3g). The La 3d spectrum of Li‐BM‐LLZTO has only the absorption peak of La_2_O_3_, and the absorption peak of metal La is not observed. The reason is that the chemical property of metal La is very active, and it will be oxidized quickly when exposed to air, to react to produce La_2_O_3_, Therefore, only the La 3d absorption peak of La_2_O_3_ can be detected in the La 3d spectrum of Li‐BM‐LLZTO. In addition, the Zr 3d and Ta 4f spectra of the BM‐LLZTO surface were analyzed (Figures S11 and S12, Supporting Information), and we found absorption peaks of ZrF_4_ and TaF_5_ on the BM‐LLZTO surface, indicating that HF indeed react with LLZTO to generate ZrF_4_ and TaF_5_. At the same time, after the BM‐LLZTO surface is etched by Ar^+^ for 15 nm, the absorption peak of Zr 3d is almost undetectable, while the absorption peak of TaF_5_ is very weak and almost nonexistent, indicating that the thickness of ZrF_4_ and TaF_5_ in BM‐LLZTO is about 15 nm, which is very small compared with the thickness of LaF_3_ and LiF (about 2 µm). This indicates that the reaction of HF with LLZTO indeed produces metal fluoride of Zr and Ta, but compared with LiF and LaF_3_, the amount of ZrF_4_ and TaF_5_ is very small. Therefore, LiF and LaF_3_ still play a major role at the interface between lithium metal and BM‐LLZTO.

**Figure 4:**
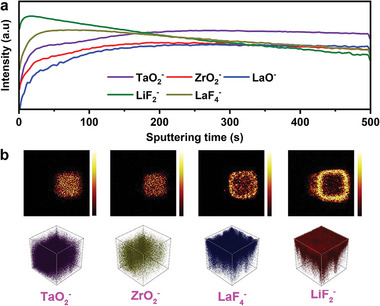
a) TOF‐SIMS depth profiles for the BM‐LLZTO pellet. b) TOF‐SIMS chemical mappings after sputtering and 3D view of element distribution.

**Figure** **4** presents the time‐of‐flight secondary‐ion mass spectroscopy (TOF‐SIMS) depth profiling of LiF and LaF_3_ on the surface of BM‐LLZTO, which reveals the evolution of several fragments as the sputtering proceeds in a negative mode. LiF_2_^−^ and LaF_4_^−^ represent LiF and LaF_3_, respectively. TaO_2_^−^, ZrO_2_^−^, and LaO^−^ signals come from LLZTO ceramic pellets. As shown in Figure 4a, the LiF_2_^−^ and LaF_4_^−^ signals began to be strong, and then gradually declined with the sputtering time. The existence of LiF and LaF_3_ on the BM‐LLZTO surface is proved, which is consistent with the results of XPS. On the contrary, the TaO_2_^−^, ZrO_2_^−^, and LaO^−^ fragments signal is very weak when it appears, increases almost in parallel with the sputtering time, and finally tends to be stable, which means that the BM‐LLZTO surface is covered with a layer of material with homogeneous element distribution. Figure 4b shows the TOF‐SIMS mapping of TaO_2_^−^, ZrO_2_^−^, LiF_2_^−^, and LaF_4_^−^ signals after sputtering and corresponding 3D visual views. It can be seen from mapping, that after sputtering for 500 s, a strong LiF_2_^−^ and LaF_4_^−^ signal can still be detected, which means that sputtering for 500 s is not enough to remove LiF and LaF_3_ from the surface. From the 3D view, the surface of BM‐LLZTO is mainly composed of LiF and LaF_3_, LiF is located at the top layer, and the interior is composed of LiF and LaF_3_.

**Figure 5:**
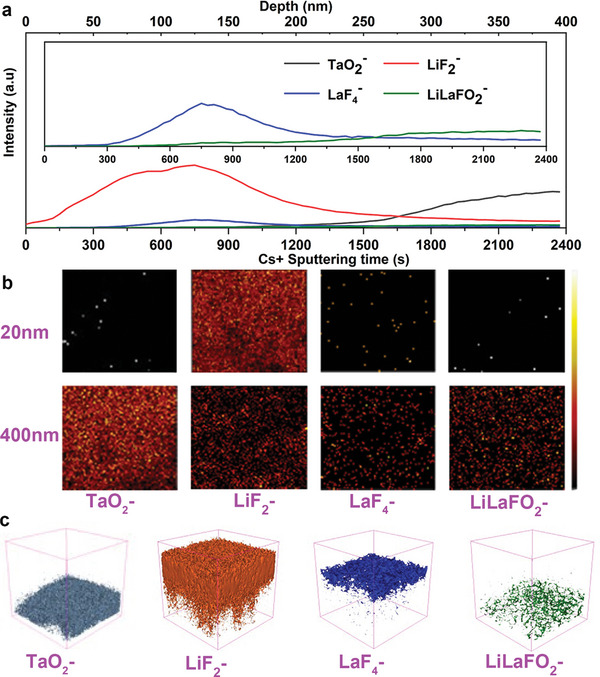
a) TOF‐SIMS depth profiles for the Li‐BM‐LLZTO pellet. b) TOF‐SIMS chemical mappings different sputtering depth. c) 3D view of element distribution.

Because TOF‐SIMS has the advantages of high sensitivity and high resolution, we still use TOF‐SIMS to further explore the diffusion mechanism of Li on the Li‐BM‐LLZTO surface. It is worth noting that Li^−^ signal comes from LiF, metal Li, Li_2_CO_3_, and LLZTO at the bottom. LiCO_3_^−^ signal comes from Li_2_CO_3_. Since there is a very thick molten Li layer on the surface of Li‐BM‐LLZTO, to obtain the surface structure information, the surface was thinned before characterization. Despite the thinning treatment, a very strong Li‐signal can still be detected on the Li‐BM‐LLZTO surface (Figure S13, Supporting Information), This is due to the presence of a large amount of metal Li on its surface. In addition, the signal of LiCO_3_^−^ can also be detected on the surface, which is due to the brief exposure of the Li‐BM‐LLZTO pellets to air, which causes the metal Li on the surface to react with CO_2_ to generate Li_2_CO_3_. This signal drops rapidly after 30 s of sputtering, indicating that the Li_2_CO_3_ is very thin, and this result is also reflected in the 2D mapping and 3D view (Figure S14, Supporting Information). It can be seen from the LiF_2_^−^ signal curve in **Figure** **5**a that, at the beginning of detection, the LiF_2_^−^ signal began to be weak because of the shielding of metal Li and Li_2_CO_3_ on the surface. The LiF_2_^−^ signal increases gradually in 0–800 s and decreases gradually in 800–2400 s. Combined with the 2D mapping and 3D view of LiF_2_^−^ (Figure 5b,c and Figure S15, Supporting Information), only a very small amount of LiF remains after sputtering for 2400 s. Due to the occlusion by metal Li, Li_2_CO_3_, and LiF, the signal of TaO_2_^−^ was only detected at 1200 s, and gradually increased from 1200 to 2400 s (Figure 5a). It can be seen from 2D mapping and 3D view (Figure 5b,c and Figure S15, Supporting Information) that LLZTO already exists at 2400 s. For LaF_3_, there is almost no LaF_3_^−^ signal within 0–300 s (Figure 5a). The reason is that the surface LaF_3_ reacts with molten lithium and converts it into LiF and metal La (reaction Equation (3)). After that, the LaF_3_^−^ signal appeared but was weak within 300–1200 s, and almost disappeared after 1200 s. Combined with the 2D mapping and 3D view of the LaF_3_^−^ signal (Figure 5b,c and Figure S15, Supporting Information), LaF_3_ only exists in a small amount in the middle layer. The reason is the insufficient reaction between LaF_3_ and molten Li. It is worth noting that the existence of the LiLaFO_2_^−^ signal proves the existence of Li_6.5−_ *_x_* La_3−_ *_y_* Zr_1.5_Ta_0.5_O_12−_ *_x_*_−_ *_y_* F*_x_*_−_ *_y_* products and explains the rationality of the chemical reaction Equation (2). It can be seen from Figure 5b,c and Figure S15, Supporting Information, that the Li_6.5−_ *_x_* La_3−_ *_y_* Zr_1.5_Ta_0.5_O_12−_ *_x_*_−_ *_y_* F*_x_*_−_ *_y_* thin layer is located at the bottom and mixed with LLZTO. Based on the above analysis, it is not difficult to understand the reasons why molten lithium has a very good wettability for BM‐LLZTO. First, the main components of the 3D‐BM structure are LaF_3_ and LiF. LaF_3_ reacts with molten lithium to generate metal La, which in turn reacts with molten lithium to generate Li—La alloy. The introduction of the La atom weakens the force of neighboring lithium atoms, reduces the surface tension of liquid lithium, and improves the wettability of liquid lithium.^[^ ^58^ ^]^ Second, LiF is a very good lithiophilic material. The surface of BM‐LLZTO contains a large amount of LiF, which greatly improves the wettability of lithium metal. Finally, the surface of 3D‐BMs has a large number of micro/nano channels, which can generate a large amount of capillary force to improve the wettability of liquid lithium.^[^ ^59^ ^]^

**Figure 6:**
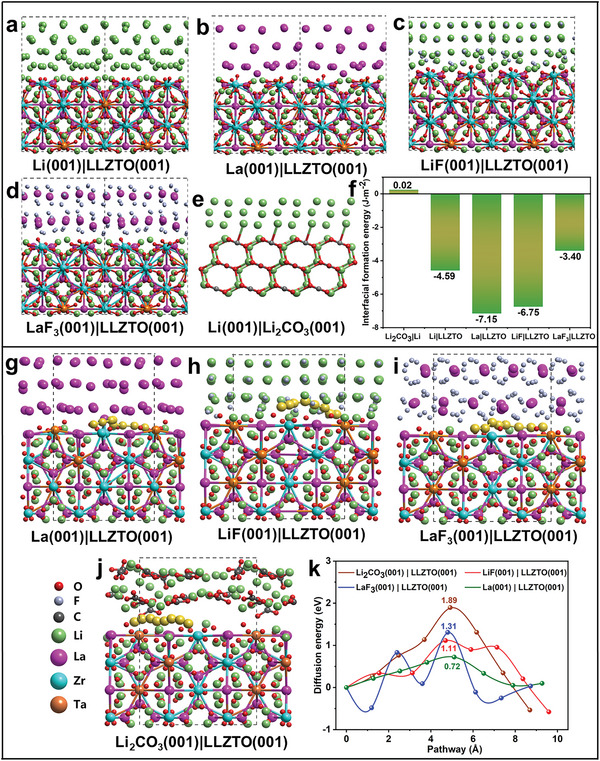
The DFT calculations of interfacial formation energies of a) Li (001)|LLZTO (001), b) La (001)|LLZTO (001), c) LiF (001)|LLZTO (001), d) LaF_3_ (001)|LLZTO (001), and e) Li (001)|Li_2_CO_3_ (001). f) Interface formation energy comparison diagram. Li migration path in g) La (001)|LLZTO (001), h) LiF (001)|LLZTO (001), i) LaF_3_ (001)|LLZTO (001), and j) Li_2_CO_3_ (001)|LLZTO (001). k) Energy profiles of the Li transfer pathways. The Li migration path is denoted as yellow balls.

The interface chemistry of Li‐LLZTO was then researched by DFT calculations. As shown in **Figure** **6**a–d, the interfacial formation energy of Li|LLZTO, La|LLZTO, LiF|LLZTO, and LaF_3_|LLZTO is −4.59, −7.15, −6.75, and −3.40 J m^−2^, respectively, which illustrate the intrinsic capabilities of these materials to wet LLZTO. This result is also confirmed in our experiment (Figure 1e). Furthermore, the interfacial formation energies of La|LLZTO and LiF|LLZTO were more negative than those of Li|LLZTO, and LaF_3_|LLZTO is slightly higher than Li|LLZTO, which shows that the production of these film layers is conducive to enhancing the chemical contact between Li and LLZTO. On the contrary, the interface formation energy of Li|Li_2_CO_3_ is positive (Figure 6e,f), which means that Li cannot wet Li_2_CO_3_, and this result was observed in our experiment (Figure 1c). Furthermore, due to the extremely high reactivity, the bare LLZTO surface will form a lithiophobic layer dominated by Li_2_CO_3_ with low lithium‐ion conductivity in the air.^[^ ^25^ ^]^ The DFT calculation results show that Li_2_CO_3_|LLZTO (Figure 6g) has a very high diffusion barrier of 1.89 eV, which may lead to the trend of lithium accumulation in the region with relatively high current density, and then bring about the growth of lithium dendrites. By contrast, the migration energy barriers of Li^+^ at La|LLZTO (Figure 6g), LiF|LLZTO (Figure 6h), and LaF_3_|LLZTO (Figure 6i) are 0.72, 1.11, and 1.31 eV respectively, and all the migration energy barriers are lower than those at Li_2_CO_3_|LLZTO. The low migration energy barriers facilitate Li^+^ transfer across the interface and the uniform distribution of Li^+^, thereupon then effectively inhibiting the growth of Li dendrites.

**Figure 7:**
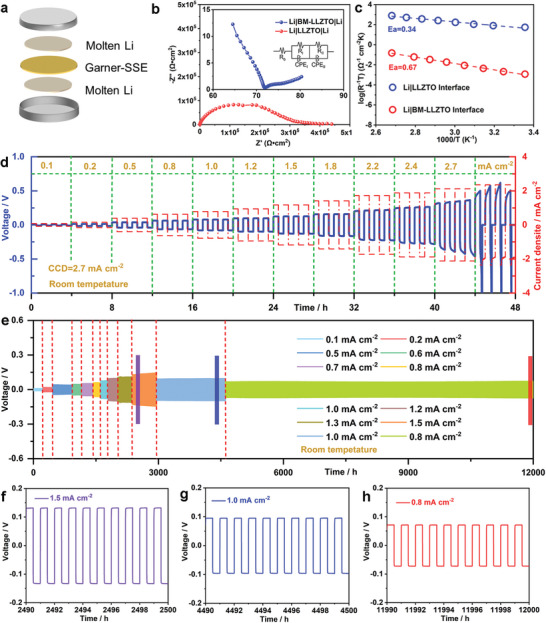
a) Schematic diagram of Li symmetric cell. b) LLZTO and BM‐LLZTO Li symmetrical cells EIS spectra. c) The activation energy (*E*_a_). d) CCD of the Li|BM‐LLZTO|Li cells. e) Galvanostatic cycling performance of the Li|BM‐LLZTO|Li cell under 0.1–1.5 mA cm^−2^ at RT. f–h) Enlarged view of the galvanostatic curve under the current density of 1.5, 1.0, and 0.8 mA cm^−2^.

Li|LLZTO interfacial performance is usually evaluated by Li symmetric cell testing (**Figure** **7**a). Figure 7b shows the EIS curves at room temperature (RT), and among them, The EIS curve of Li|LLZTO|Li presents a semicircle. The starting point is the resistance of the LLZTO ceramic pellets. Considering that a symmetric cell has two charge transfer interfaces, half of the semicircle value represents the interface resistance of one interface. Therefore, the interface resistance of Li|LLZTO|Li is 198956 Ω cm^2^. Surprisingly, the interface resistance of Li|BM‐LLZTO|Li is only 3 Ω cm^2^, and the total resistance is only 78 Ω cm^2^. This shows that the interface resistance between Li|LLLZTO is reduced by about five orders of magnitude after 40% HF solution treatments. In addition, temperature also has an important influence on the interface resistance, at 25–100 °C, the interfacial resistance of Li|LLZTO, and Li|BM‐LLZTO conforms to Arrhenius behavior (Figure 7c). The activation energy of the Li|LLZTO interface is 0.67 eV, while that of Li|BM‐LLZTO is only 0.34 eV. The very low activation energy implies a high Li^+^ transport rate and a low Li^+^ migration barrier at the Li|BM‐LLZTO interface, which is conducive to the rapid passage of ions through the interface layer and therefore improves the rate performance. The maximum withstands current density of a Li battery without battery failure in a solid‐state Li metal battery is generally defined as the CCD. Therefore, the CCD is an important parameter for the application of solid‐state Li metal batteries, which helps to determine the rate‐determining steps of the lithium kinetics of solid‐state batteries.^[^ ^60^ ^]^ The step‐type constant current charging and discharging method is the most common method for testing CCD. In this work, the current density of CCD is tested from 0.1 mA cm^−2^ until the symmetrical battery is short‐circuited. At room temperature, Li|LLZTO|Li has a large fluctuation in voltage at a current density of 0.1 mA cm^−2^, indicating that its CCD is around 0.1 mA cm^−2^ (Figure S16, Supporting Information). In contrast, the voltage curve of Li|BM‐LLZTO|Li is very stable, and its CCD is as high as 2.7 mA cm^−2^ (Figure 7d). As far as we know, the CCD of 2.7 mA cm^−2^ is one of the highest values in the garnet solid electrolyte (Table S4, Supporting Information). The reasons for the great improvement of CCD are as follows. First, a high concentration of HF solution will react with Li_2_CO_3_ and LLZTO to generate LiF—LaF_3_ with an ionic conductor‐type 3D‐BM structure on the surface. Second, Due to the presence of F vacancies in LiF, when reacted with Li metal, F vacancies can increase the carrier concentration and mobility, resulting in high Li^+^ conductivity in LiF.^[^ ^61^ ^]^ Meanwhile, LaF_3_ has high lithium‐ion conductivity, which can reach 10^−5^ S cm^−1^.^[^ ^62^ ^]^ The high ionic conductivity of LiF and LaF_3_ can effectively enhance the mobility of Li^+^ at the Li|LLZTO interface. Third, the 3D‐BM structure has a very large specific surface area, and the wettability and diffusivity of molten Li can be improved by capillary force, which can effectively reduce the local current and the volume change of the Li anode.^[^ ^59^ ^]^ Fourth, LaF_3_ reacts with molten Li to form metallic La and LiF. Metallic La and molten Li form an electronic conductor Li—La alloy. Li—La alloy can guide lithium nucleation and growth, and greatly improve the CCD of the battery. In addition, the production of Li—La alloy will transform the ionic conductor of the 3D‐BM structure into the mixed ionic electronic conductor of the 3D‐BM structure (3D‐MIEC). The large surface area of 3D‐MIEC facilitates the kinetic transport of Li^+^ and electrons, and provides sufficient space for the deposition of Li atoms, reducing the generation of interfacial stress.^[^ ^63^ ^]^ Finally, the low surface energy and diffusion energy are favorable for the uniform deposition of Li^+^ and the uniform distribution of the electric field, which inhibits the growth of Li dendrites. The galvanostatic cycling experiment is used to detect the stability of the Li|LLZTO interface and the ability to inhibit Li dendrites. The Li|LLZTO|Li symmetrical battery has a very large voltage at room temperature and 0.1 mA cm^−2^ current density, and it is short‐circuited after only one cycle (Figure S16, Supporting Information). This is because Li_2_CO_3_ formed on the LLZTO surface has a very poor wettability with Li metal, resulting in uneven Li^+^ deposition. In contrast, Li|BM‐LLZTO|Li shows very excellent interface performance. The polarization voltages at 0.1, 0.2, 0.5, 0.6, 0.7, 0.8, 1.0, 1.2, 1.3 and 1.5 mA cm^−2^ are 7.9, 16.7, 40.3, 45.2, 51.7, 59.2, 76.8, 95.7107.8, and 128.4 mV, respectively (Figure 7f–h and Figure S17, Supporting Information), and the polarization voltage under different current densities remains stable and cycling over 12 000 h (Figure 7e). At the same time, the impedance calculated by Ohm's law under different current densities is close to the actual measured impedance value of 78 Ω cm^2^ (Figure 7b), indicating that the battery is working well and there is no short circuit. To better reflect the inhibition performance of BM‐LLZTO on Li dendrites for a long time, after a long cycle at a current density of 1.5 mA cm^−2^, the current density is adjusted to 1.0 and 0.8 mA cm^−2^ for a long cycle again, and the polarization voltages are 91 and 68 mV respectively, only slightly higher than the previous polarization voltage at 1.0 and 0.8 mA cm^−2^. At the same time, Li|BM‐LLZTO|Li can still be stably cycled for 30 h at a high capacity of 5 mAh cm^−2^ (Figure S18, Supporting Information).

**Figure 8:**
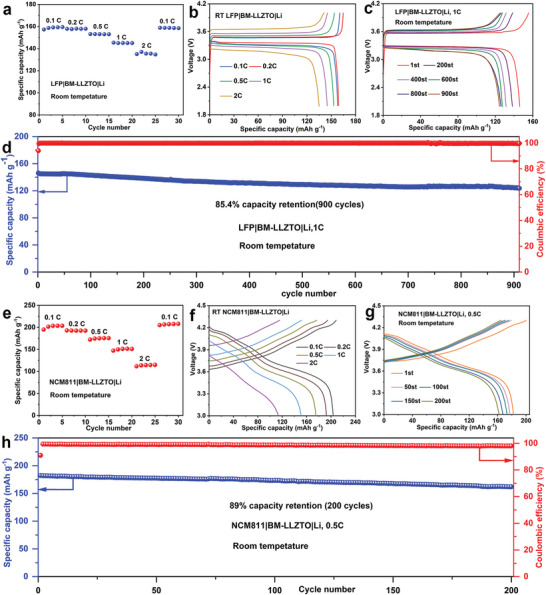
a,b) Rate performance of the LFP|BM‐LLZTO|Li. c,d) Cycle performance of the LFP|BM‐LLZTO|Li cell under 1 C at room temperature. e,f) Rate performance of the NCM811|BM‐LLZTO|Li. g,h) Cycle performance of the NCM811|BM‐LLZTO|Li cell under 0.5 C at room temperature.

To illustrate the feasibility of BM‐LLZTO in an all‐solid‐state battery, lithium metal was used as the negative electrode and LFP as the positive electrode to assemble the full battery. To solve the problem of poor wettability between the LFP cathode material and the LLZTO solid electrolyte, 10 µL liquid electrolyte was dropped between the LFP cathode material and the LLZTO solid electrolyte to improve the interface problem between LFP|LLZTO. As shown in **Figure** **8**a, the initial discharge capacity of LFP|BM‐LLZTO|Li at 0.1 C is 159.5 mAh g^−1^. The discharge specific capacity under 0.2 C, 0.5 C, 1 C, and 2 C are 158.0, 153.1, 145.4, and 135.5 mAh g^−1^ (Figure 8a,b), respectively. After high‐rate cycling, the battery's capacity under 0.1 C can be restored to 158.9 mAh g^−1^, realizing a capacity recovery rate of up to 99.7%. Cycle performance is battery life, and the importance of cycle performance is needless to say. At room temperature, LFP|BM‐LLZTO|Li can cycle stably for 900 cycles at a 1 C rate, and the capacity retention rate reaches 85.4% (Figure 8c,d), showing extremely excellent cycle performance. Remarkably, the Coulombic efficiency of the solid‐state battery is up to 99.9% (Figure 8d), which is due to the distinguished interface performance of BM‐LLZTO. The high Coulombic efficiency also indicates that there is neither lithium dendrite formation nor interface side reaction during the cycle of the battery. As far as we know, the battery performance of LFP|BM‐LLZTO|Li is also the best (Table S5, Supporting Information). The battery's high capacity retention, long cycle performance, and excellent rate performance benefit from good interface wetting, high lithium‐ion migration, and a dendrite‐free interface. Moreover, BM‐LLZTO solid electrolyte pellets are also combined with NCM811 cathode material to assemble the NCM811|BM‐LLZTO|Li battery. As shown in Figure 8e, the initial capacity of the battery at 0.1 C is 203.5 mAh g^−1^, after high‐rate cycles of 0.2 C, 0.5 C, 1 C, and 2 C, the specific capacities are 193, 175.6, 151.5, and 114.8 mAh g^−1^, respectively (Figure 8f), the battery shows very excellent rate performance. When the battery's rate returns to 0.1 C, its specific capacity is 208 mAh g^−1^. The specific capacity does not decay, showing extremely excellent recovery performance. As Figure 8g illustrates, at room temperature, NCM811|BM‐LLZTO|Li can cycle stably for 200 cycles at 0.5 C rate, and the capacity retention and Coulombic efficiency are up to 89% and 98.6% (Figure 8g,h), respectively. This indicates that the cell of NCM811|BM‐LLZTO|Li has an outstanding cycle performance, which provides great feasibility for its practical application. In Figure S19, Supporting Information, NCM811|BM‐LLZTO|Li and LFP|BM‐LLZTO|Li battery can light LED bulb, and BM‐LLZTO has a great application prospect in a solid‐state battery.

## 3 Conclusion

In a summary, a superlithiophilic 3D‐BM interface layer composed of ionic conductor LiF—LaF_3_ was constructed on the Li|LLZTO interface to achieve a high‐rate and ultra‐stable solid‐state lithium metal battery. The Li|BM‐LLZTO|Li cell shows a very low interfacial resistance (3 Ω cm^2^), a high CCD of 2.7 mA cm^−2^, and a stable operation for more than 12 000 h in Li plating/stripping cycles. At room temperature, the LFP|BM‐LLZTO|Li full battery has a capacity retention of 85.4% after 900 cycles and a high rate capacity of 135.5mAh g^−1^ at 2 C. In addition, the NCM811|BM‐LLZTO|Li battery shows capacity retention of up to 89% after 200 cycles at 0.5 C. The roles of the 3D‐BM interface layer were also clarified by a combination of experimental techniques and calculations. In addition, it is worth noting that BM‐LLZTO also shows an excellent storage performance, and no Li_2_CO_3_ is regenerated after 90 days of storage in air. This work provides a facile strategy to effectively solve the interface issues on Li|LLZTO, and also shows the feasibility of the development of solid‐state batteries with high safety, high cycle stability, and high rate performance.

## 4 Experimental Section

### LLZTO Synthesis

Synthesis of Li_6.5_La_3_Zr_1.5_Ta0.5O_12_ (LLZTO) solid electrolyte by high‐temperature solid‐phase method. LiOH (99%, with 10% excess to compensate for the sintering loss), ZrO_2_ (99%), La_2_O_3_ (99%), Ta_2_O_5_ (99%) with stoichiometry ratio was used as raw materials. Raw materials were thoroughly mixed at 400 rpm for 10 h in a planetary ball mill with zirconium oxide balls and isopropanol (IPA) as the grinding media. The mixture was dried at 80 °C for 12 h and subsequently calcined at 950 °C for 6 h in a magnesium oxide crucible. After calcined, LLZTO powder is milled (400 rpm, 10 h) and dried (80 °C, 12 h) again. The LLZTO powder is pressed into pellets with a diameter of 13 mm. The pellets are placed in a magnesium oxide crucible and a small amount of mother powder is buried, sintered at 1320 °C for 20 min to obtain LLZTO solid electrolyte pellets. Sanding and polishing the LLZTO solid electrolyte pellets on 400 mesh sandpaper to a thickness of 0.8∼1 mm. The polished LLZTO pellets are placed in a glove box filled with argon for use.

### BM‐LLZTO Synthesis

40% HF solution was dropped onto the LLZTO surface for a different time, and then immediately transferred it to 80 °C for drying. It was taken out and the same was done on the other side. The BM‐LLZTO solid electrolyte pellets treated with 40% HF solution were transferred to a glove box filled with argon for use.

### Symmterical Cell Assembly: Li|BM‐LLZTO|Li cell

The lithium metal was placed in a stainless steel crucible and it was heated at 240 °C for 30 min to completely melt the lithium metal. The BM‐LLZTO sheet was placed in a stainless steel crucible, so that the molten lithium was completely coated on both sides of the BM‐LLZTO sheet, followed by sealing in CR2025 coin cells by applying a pressure of 500 psi.

### Symmterical Cell Assembly: Li|LLZTO|Li cell

Since molten lithium cannot be pasted on the surface of LLZTO, lithium foil was used for symmetrical battery assembly. The as‐prepared Li|LLZTO|Li was placed in CR2025 coin cells by applying a pressure of 500 psi, after that, the assembled symmetrical battery was placed in an oven at 140 °C for 48 h and then tested.

### Full Cell Assembly

The different steps in full cell assembly were given in the following sections.

### Preparation of Binder Slurry

PVDF and NMP were weighed at a mass ratio of 3%, and then were stirred at room temperature for 12 h to obtain a uniform and clear slurry for use.

### LFP/NCM811 Cathode Preparation

The active material LiFePO_4_/NCM811 was mixed with Super P, KS‐6, and PVDF (80:5:5:10 by weight), and was mixed in a homogenizer at 2200 r min^−1^ for 10 min. The resulting slurry was coated on aluminum foil and then dried at 90 °C for 20 min. After that, it was cut into discs with a diameter of 10 mm and vacuum‐dried at 110 °C for 12 h. The loading capacity of the active material is 3–4 mg cm^−1^.

### Synthesis of Positive Electrode Wetting Agent

To have better interface performance between LLZTO and positive electrode material, a very small amount of positive electrode wetting agent was added between LLZTO and the positive electrode. The positive electrode wetting agent in this work was 1.0 mol L^−1^ LiPF6 in the ethylene carbonate and diethyl carbonate (1:1 v/v).

### LFP|BM‐LLZTO|Li Cell

LFP was the cathode electrode and molten lithium was the anode electrode. 10 µL of liquid electrolyte was added between LFP and BM‐LLZTO to wet the interface. The LFP|BM‐LLZTO|Li full battery was assembled in an argon glove box.

### NCM811|BM‐LLZTO|Li Cell

NCM811 was the cathode electrode and molten lithium was the anode electrode. 10 µL of liquid electrolyte was added between NCM811 and SUD‐LLZTO‐BM to wet the interface. The NCM811|BM‐LLZTO|Li full battery was assembled in an argon glove box.

### Statistical Analysis

First‐principle calculations were performed by the DFT using the Vienna ab initio Simulation Package. The generalized gradient approximation with the Perdew–Burke–Ernzerhof functional^[^ ^2^, ^3^, ^4^was used to describe the electronic exchange and correlation effects. Uniform G‐centered k‐points meshes with a resolution of 2*π* × 0.04 Å^−1^ and Methfessel–Paxton electronic smearing were adopted for the integration in the Brillouin zone for geometric optimization. The simulation was run with a cutoff energy of 500 eV throughout the computations. These settings ensure convergence of the total energies to within 0.1 meV per atom. Structure relaxation proceeded until all forces on atoms were less than 1 meV Å^−1^ and the total stress tensor was within 0.01 GPa of the target value.

## Conflict of Interest

The authors declare no conflict of interest.

## Supporting information

Supporting Information

## Data Availability

The data that support the findings of this study are available from the corresponding author upon reasonable request.
