# Supplementary material for: Constructing a Superlithiophilic 3D Burr‐Microsphere Interface on Garnet for High‐Rate and Ultra‐Stable Solid‐State Li Batteries
Source: Adv Sci (Weinh). 2023 Feb 15;10(11):2207056. doi: 10.1002/advs.202207056 (PMC10104650; doi:10.1002/advs.202207056)
Supplement: Supplementary file 1 — Supporting Information [file ADVS-10-2207056-s001.pdf]

ADVANCED SCIENCE
Open Access

Supporting Information

for *Adv. Sci.*, DOI 10.1002/advs.202207056

Constructing a Superlithiophilic 3D Burr-Microsphere Interface on Garnet for High-Rate and Ultra-Stable Solid-State Li Batteries

*Butian Chen, Jicheng Zhang, Tianran Zhang, Ruoyu Wang, Jian Zheng, Yanwu Zhai and Xiangfeng Liu**

Supporting Information

Constructing a Superlithiophilic 3D Burr-Microsphere Interface on Garnet for High-Rate and Ultra-Stable Solid-State Li Batteries

Butian Chen[a], *Jicheng Zhang*[a], *Tianran Zhan*[a], *Ruoyu Wang*[a], *Jian Zheng*[a], *Yanwu Zhai*[a], *and Xiangfeng Liu*[ab]*

[a] Center of Materials Science and Optoelectronics Engineering, College of Materials Science and Optoelectronic Technology, University of Chinese Academy of Sciences, Beijing 100049, P. R. China

[b] CAS Center for Excellence in Topological Quantum Computation, University of Chinese Academy of Sciences, Beijing 100190, P. R. China

*Corresponding Author: liuxf@ucas.ac.cn. (X.L.) Tel. +86 10 8825 6840

1. Material Characterizations

X-ray diffraction (XRD) was performed on a Regaku smart lab (Rigaku Corporation, Japan) advanced diffractometer using a filtered Cu Ka1 radiation source (λ =1.5406Å) at 40 kV and 40 mA. XRD data were collected at the room temperature by step scanning in the 2θ range of 10 to 80°.relative densities of the LLZTO pellets were analyzed by Archimedes' method. The morphologies were characterized by field emission scanning electron microscopy (FESEM) on SU8010 (Hitachi Co., Ltd, Japan). X-ray photoelectron spectroscopy (XPS) measurement was performed on an Axis Supra (Shimadzu Corporation, Japan) using monochromatic Al K alpha (energy 1486.6 eV) X-ray source. Raman spectra were collected on a Via Renishaw Raman spectrometer system (Renishaw, UK) using a 532 nm wavelength laser. The TOF-SIMS test was conducted using TOF-SIMS IV (ION-TOF GmbH, Germany) with a 30 keV bismuth liquid metal ion source and a base pressure at $\approx 10^{-8}$ bar in the analysis chamber. The negative secondary ions were induced by the primary ion beam bombardment on the surface of LLZTO. The analysis time was 10 min. Depth profiles were obtained by sputtering ion beams of Cs+ (3 keV) on a 400 μm × 400 μm square. The sputtering rate was obtained on a Si wafer as10 nm min^{-1} with a sputtered area of 400 μm × 400 μm.

2. Electrochemical measurements

To test the ionic conductivity of the LLZTO pellets, Au films will be sputtered on both sides of the LLZTO pellets, and Au/LLZTO/Au blocking batteries will be assembled. Electrochemical impedance spectroscopy (EIS) was obtained on Solartron 1260 impedance analyzer with a voltage amplitude of 10 mV and frequency of from 1 MHz to 0.1 Hz. The calculation formula of its ionic conductivity: σ=L/(RA), where R is total impthe edance exhibited in the EIS plot, L is the thickness of the LLZTO pellets, A is the effective area between LLZTO pellets and blocking electrode Au.

3. Calculation method

First-principle calculations were performed by the density functional theory (DFT) using the Vienna Ab-initio Simulation Package (VASP) package[1]. The generalized gradient approximation (GGA) with the Perdew−Burke−Ernzerhof (PBE) functional[2-4] was used to describe the electronic exchange and correlation effects. Uniform G-centered k-points meshes with a resolution of 2π*0.04 $Å^{-1}$ and Methfessel-Paxton electronic smearing were adopted for the integration in the Brillouin zone for geometric optimization. The simulation was run with

a cutoff energy of 500 eV throughout the computations. These settings ensure convergence of the total energies to within 0.1 meV per atom. Structure relaxation proceeded until all forces on atoms were less than 1 meV Å $^{-1}$ and the total stress tensor was within 0.01 GPa of the target value. The energy barrier for Li migration was calculated using the nudged elastic band (NEB) method[5-6]. The calculation parameters and convergence criteria were kept the same as in the ground state calculations.

The interface formation energies of Li(001)|LLZTO(001),La(001)|LLZTO(001), LiF(001)|LLZTO(001), and LaF_3(001)|LLZTO(001) were evaluated by the energy difference between an interface system and the bulk energy of the two materials that comprise it, i.e., $\Delta E_F = (E_{ab}-N_a*E_a-N_b*E_b)/2S$, where E_{ab} denotes the total energy of the complete system containing the interface and depends on how many formula units of materials a and b comprise the interface (N_a and N_b, respectively), E_a and E_b are the bulk energy per formula unit for materials a and b, respectively, and S refers to the interfacial area, 2 means two interfaces in the interface models.

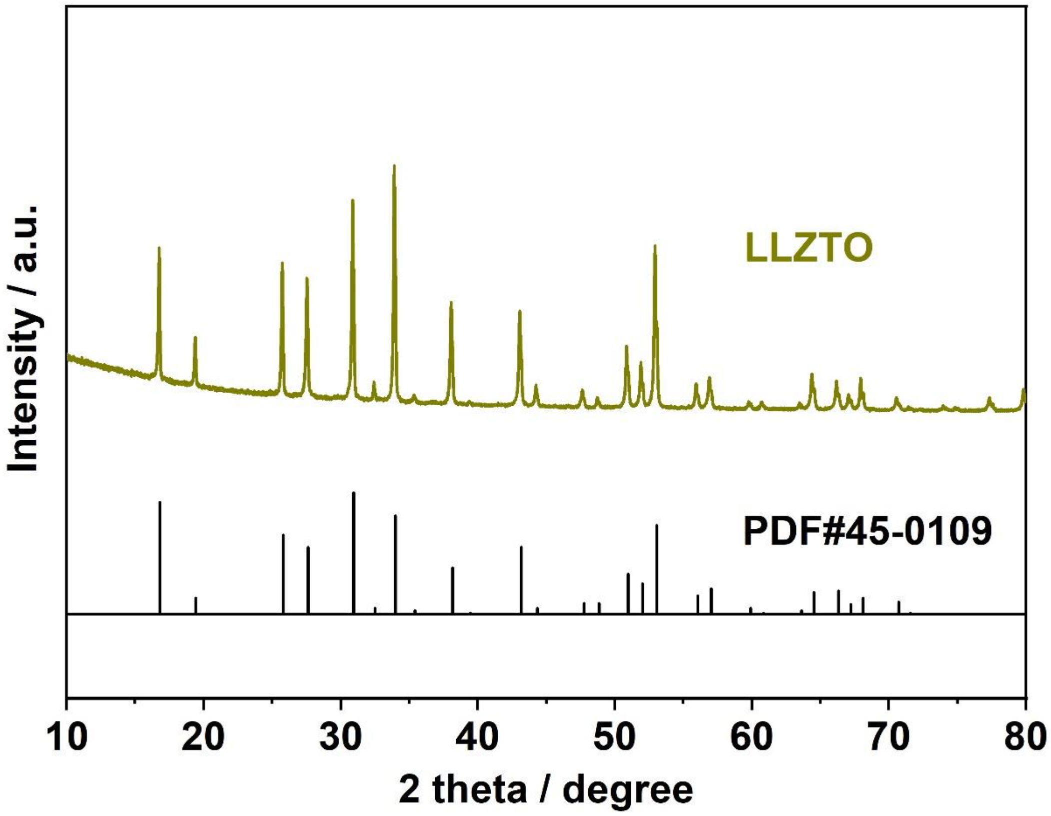

Figure S1. XRD pattern of as-synthesized LLZT pellet.

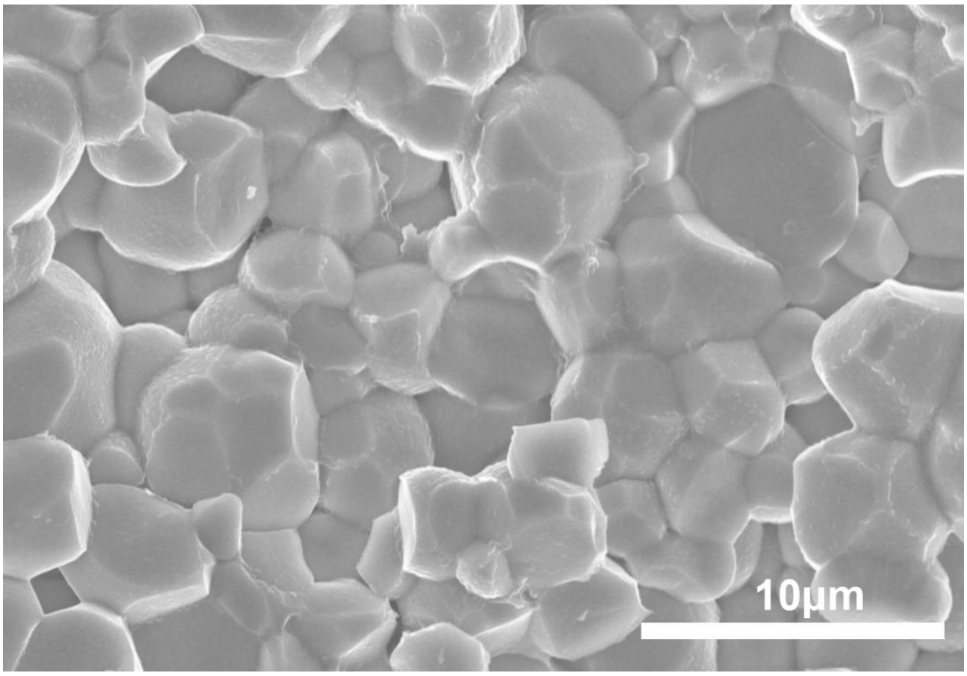

Figure S2. Cross-sectional SEM images of LLZT pellet

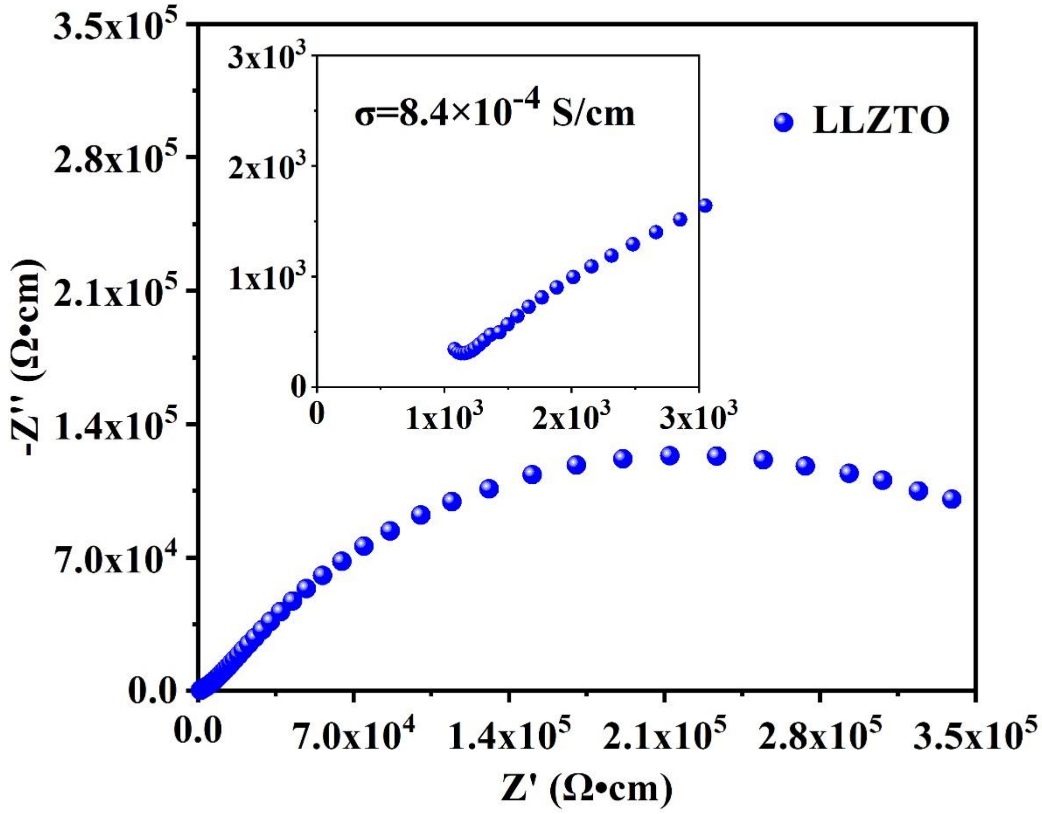

Figure S3. Ionic conductivity

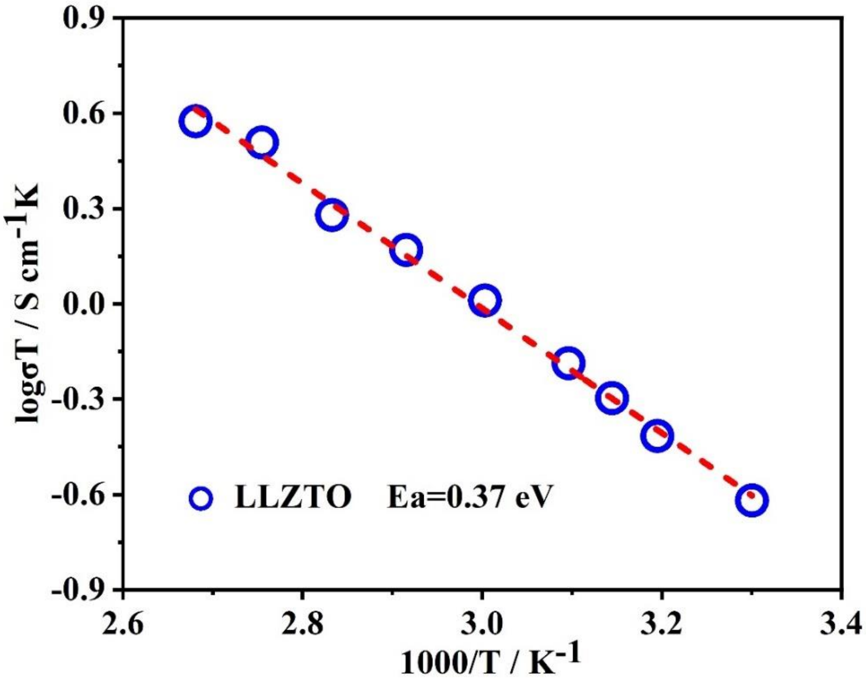

Figure S4. activation energy *Ea* of LLZT measured by EIS in the temperature from 25°C to 100 °C

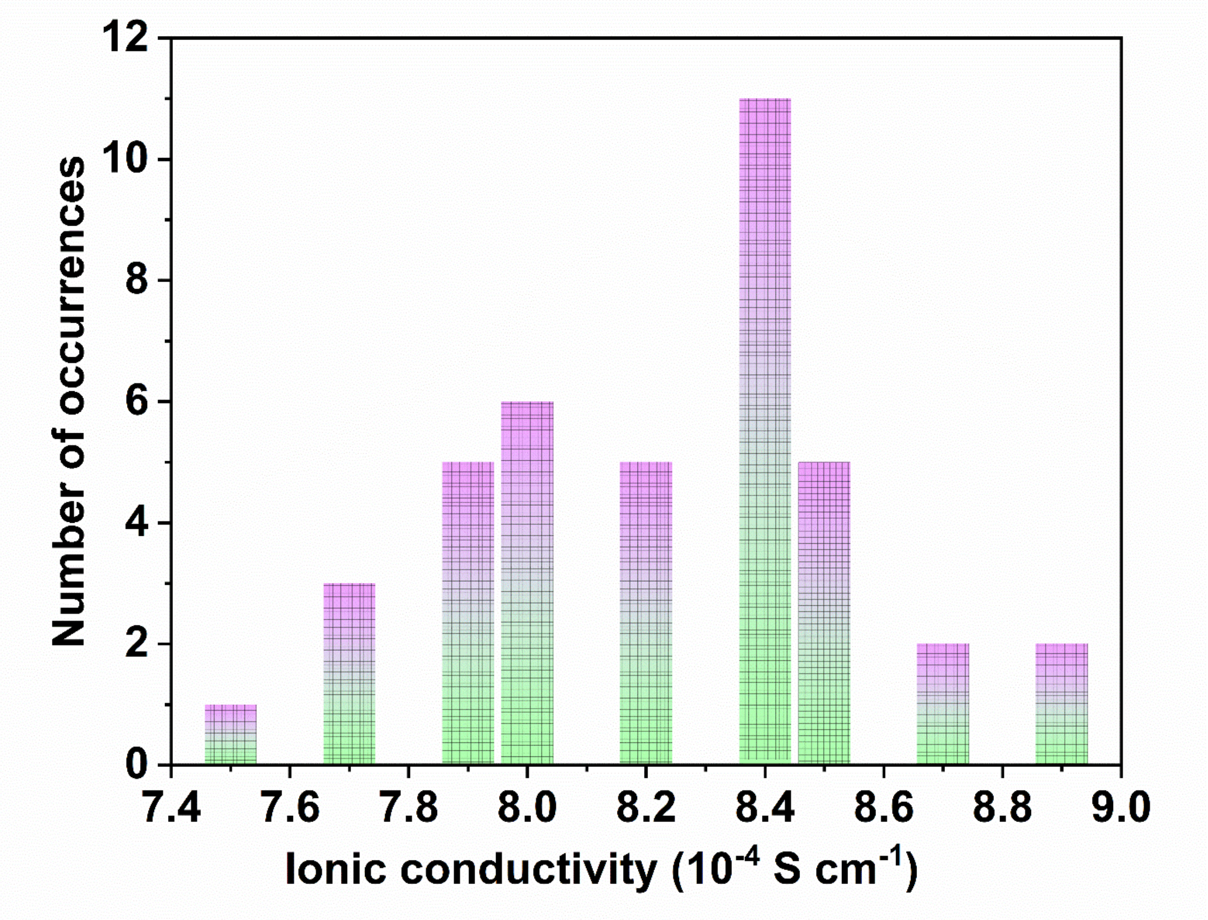

Figure S5. Ionic conductivity distribution

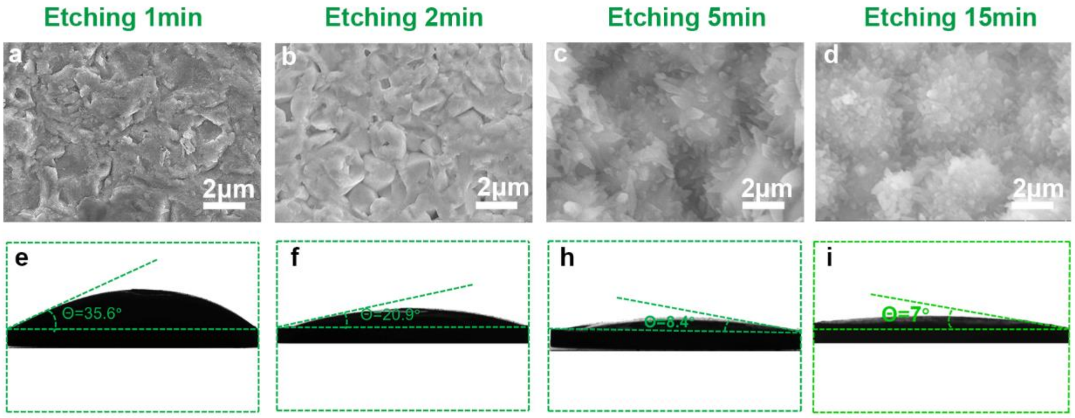

Figures S6. The SEM images of BM-LLZTO surface and the contact angle between BM-LLZTO and molten lithium were etched by HF at different times.

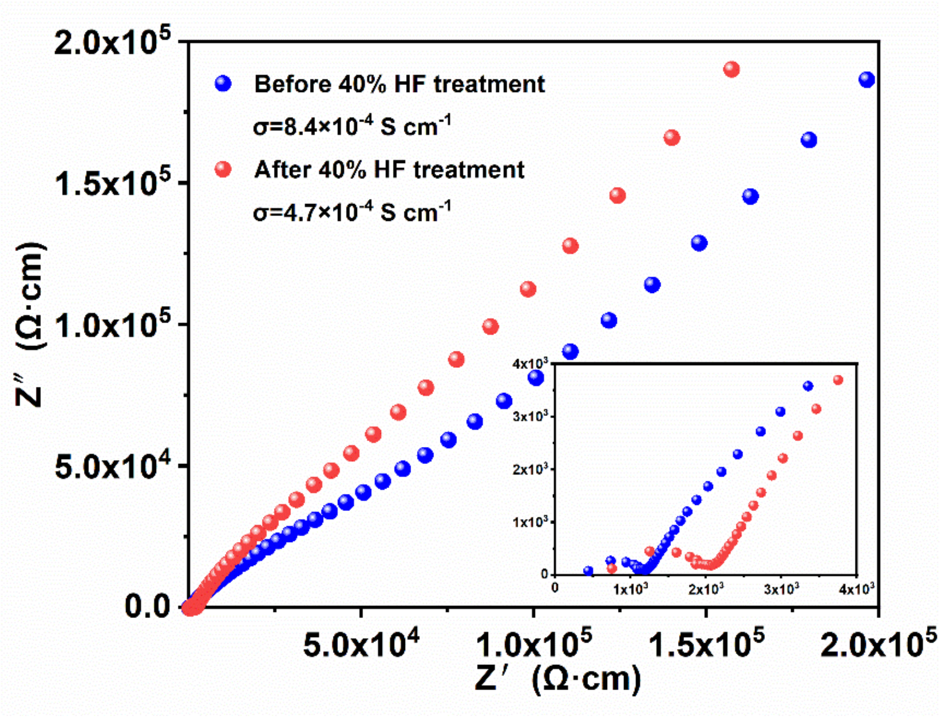

Figure S7. EIS spectra before and after HF treatment.

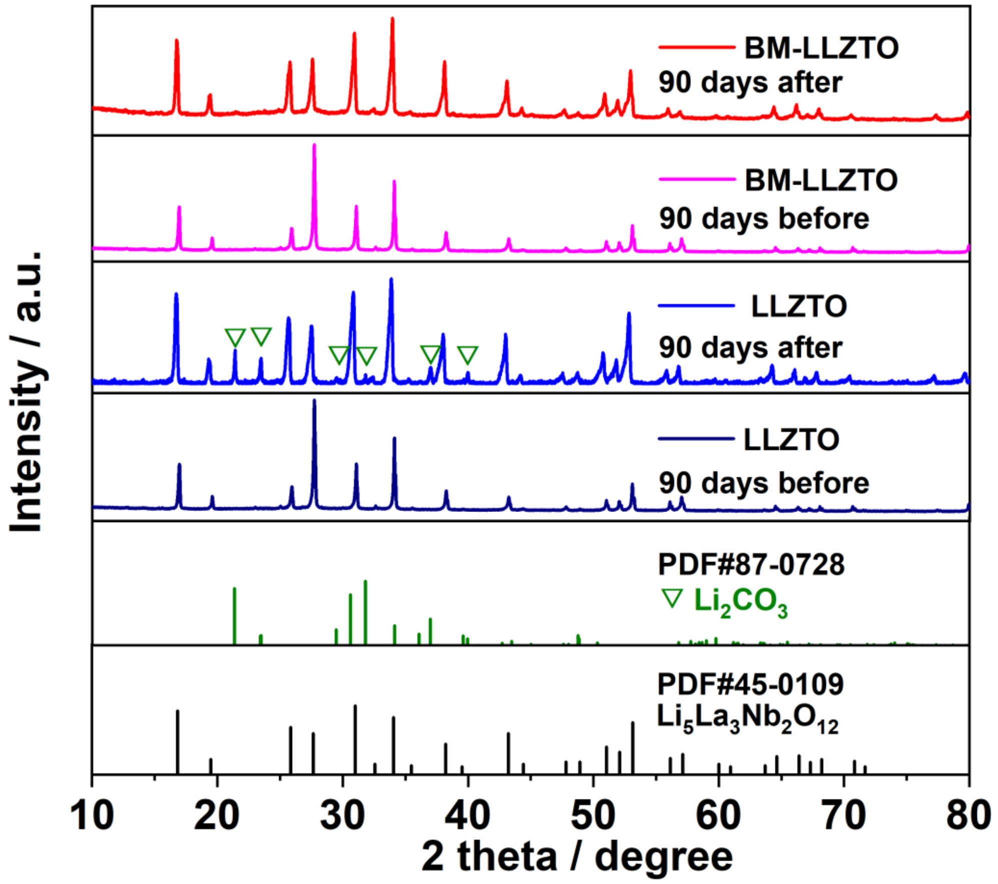

Figure S8. XRD pattern of LLZT and BM-LLZTO

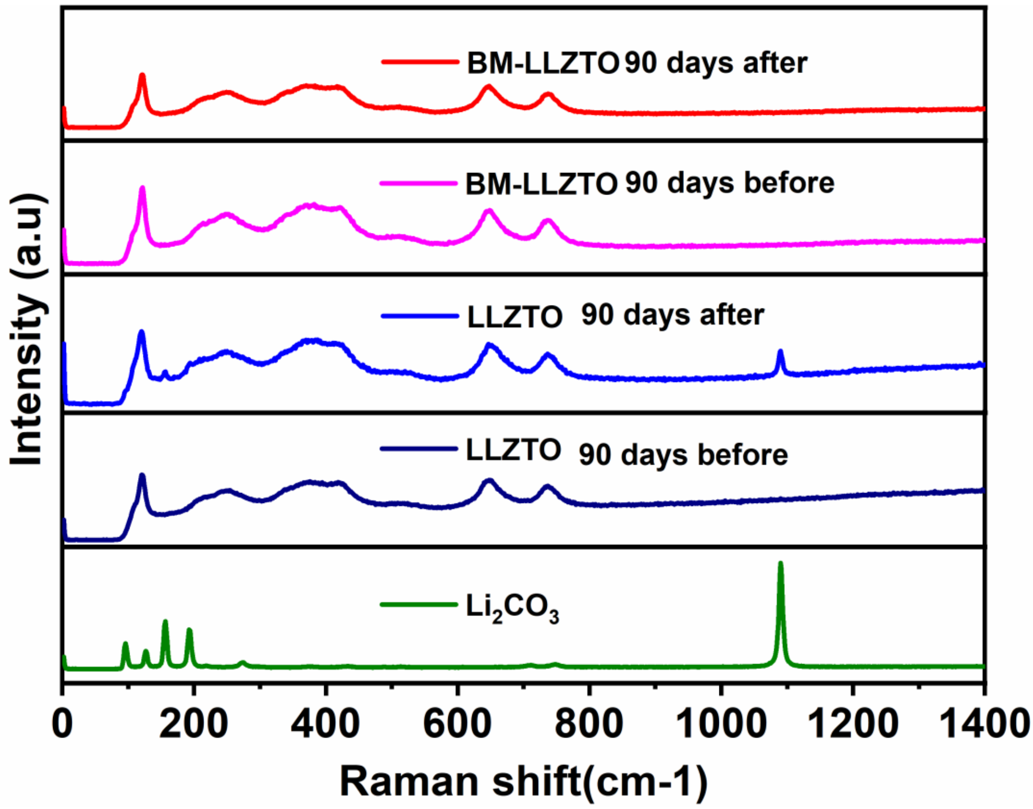

Figure S9. Raman pattern of LLZT and BM-LLZTO

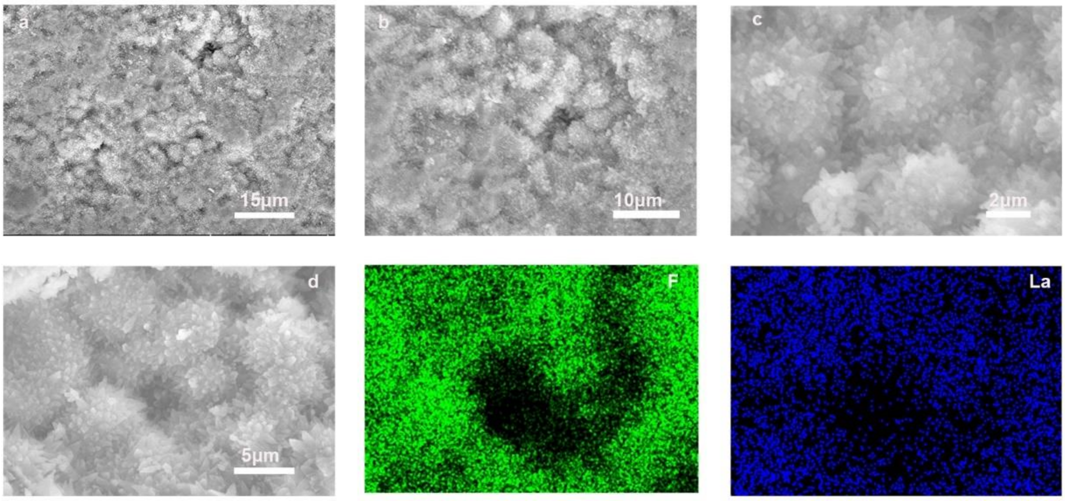

Figure S10. a-c) SEM images of BM-LLZTO surface at different magnifications.
d) EDS images of LLZTO surface

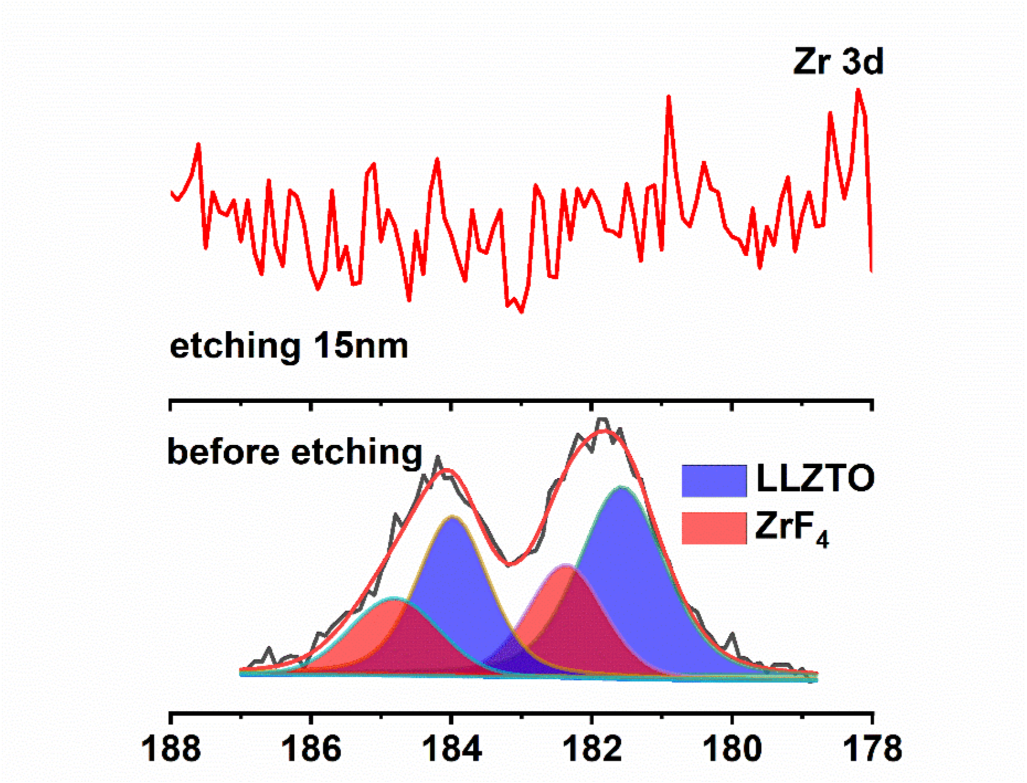

Figures S11. XPS Zr 3d spectra of BM-LLZTO surface before and after etching at 15nm

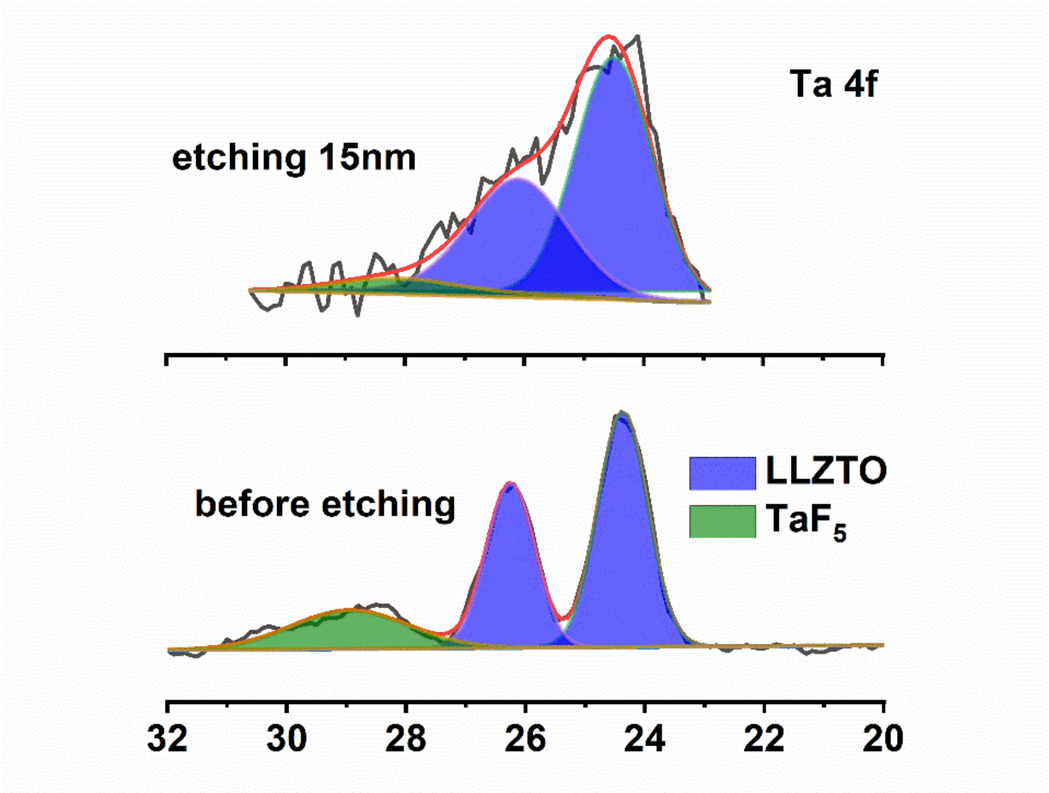

Figures S12. XPS Ta 4f spectra of BM-LLZTO surface before and after etching at 15nm

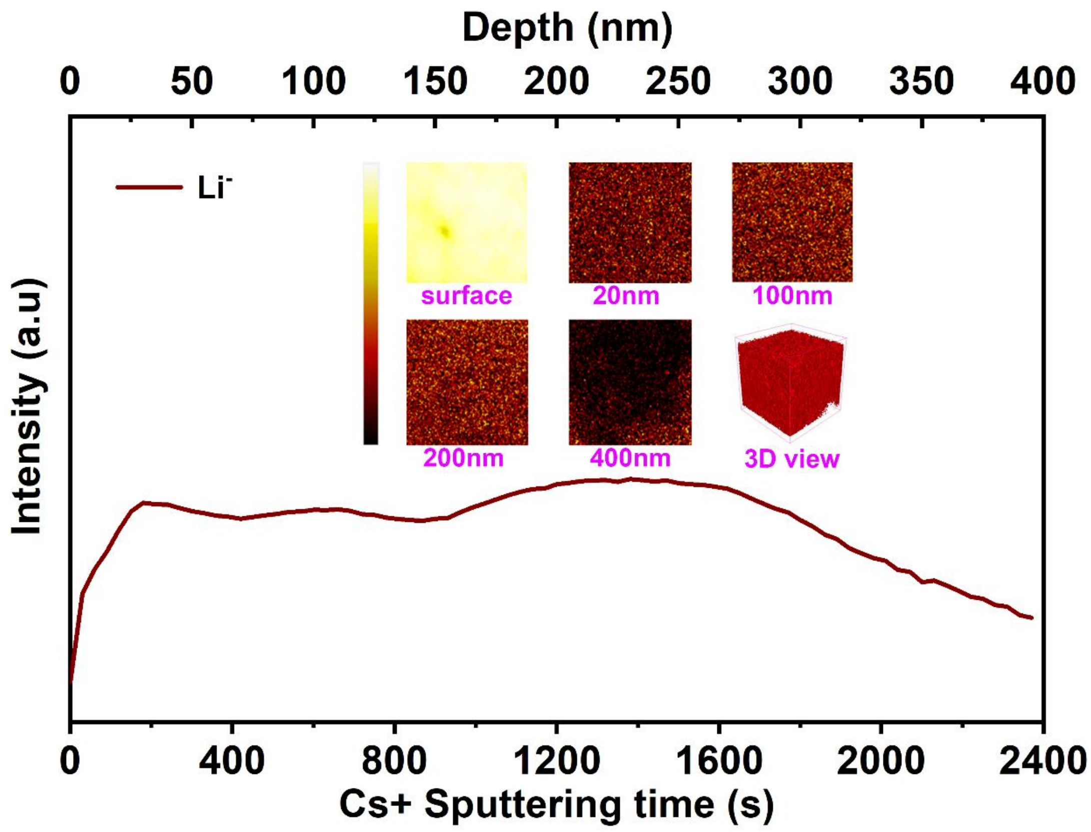

Figure S13. Depth profiles, 2D mapping, and 3D view of the $LiCO_3^-$ signal

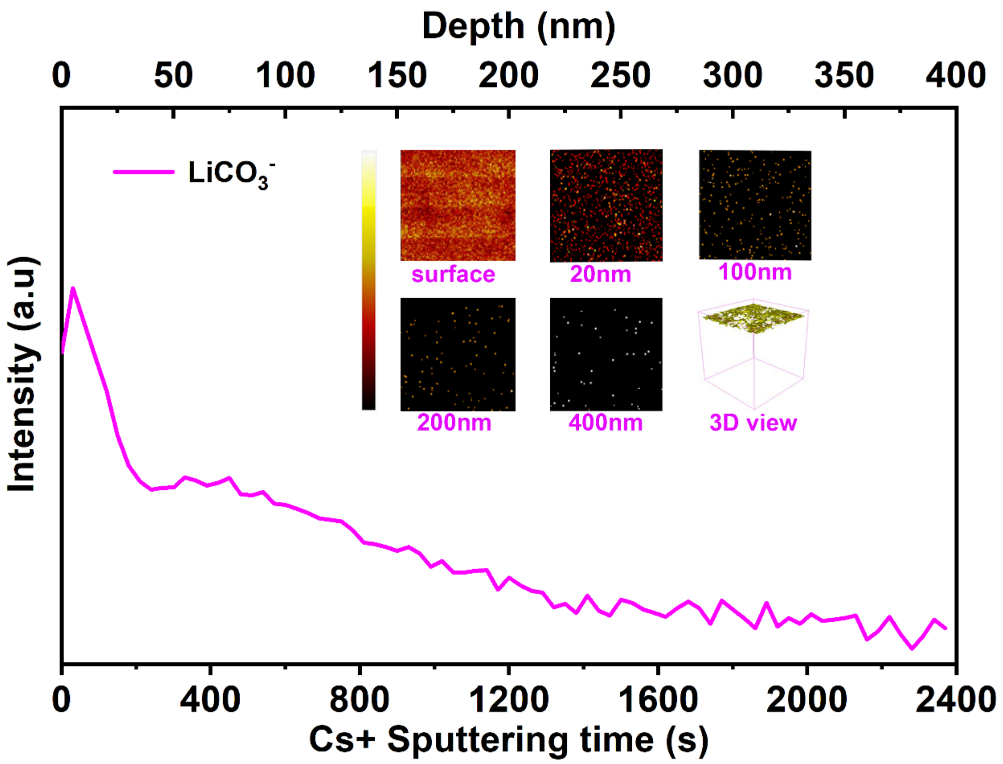

Figure S14. Depth profiles, 2D mapping, and 3D view of the Li^- signal

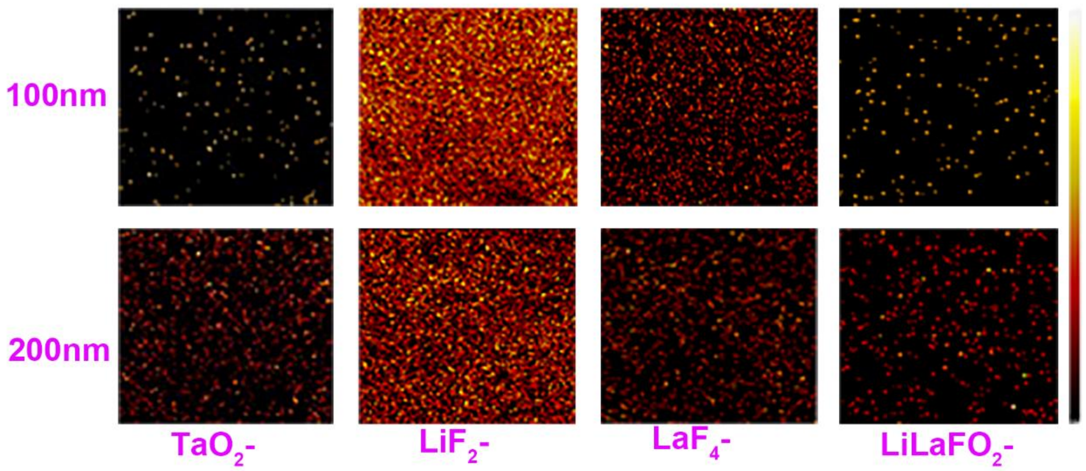

Figure S15. 2D mapping of the TaO_2^- ,LiF_2^-,LaF_4^- and $LiLaFO_2^-$ signal

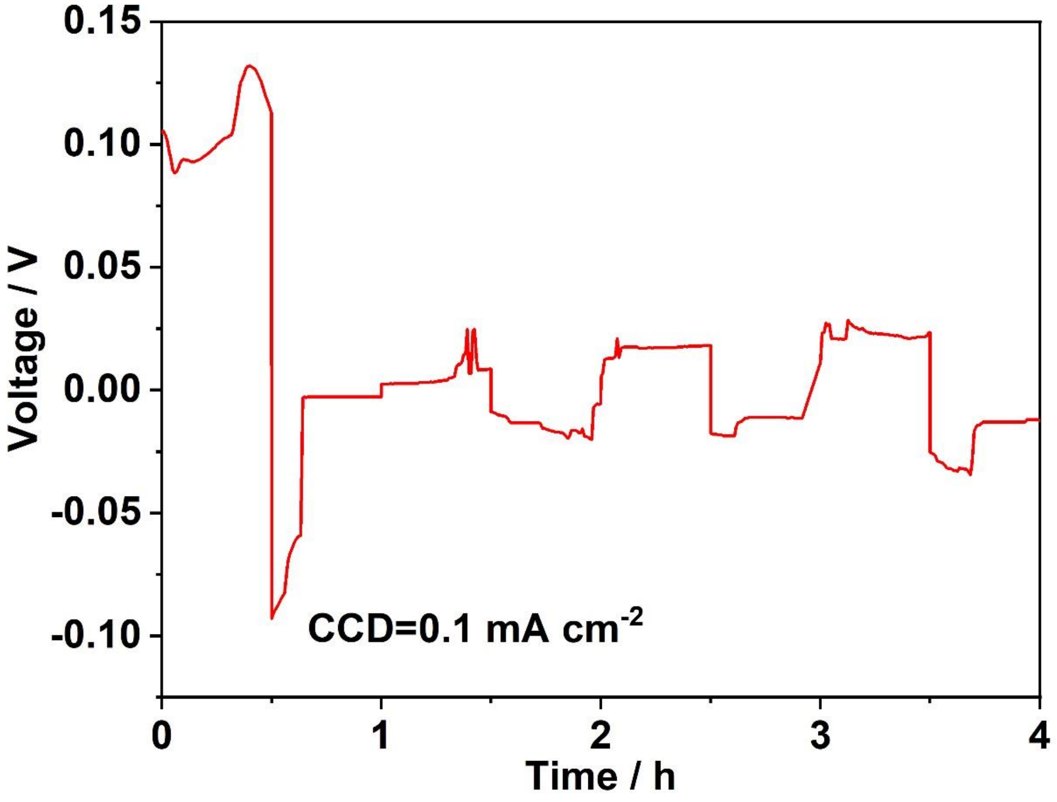

Figure S16. The CCD diagram of LLZTO

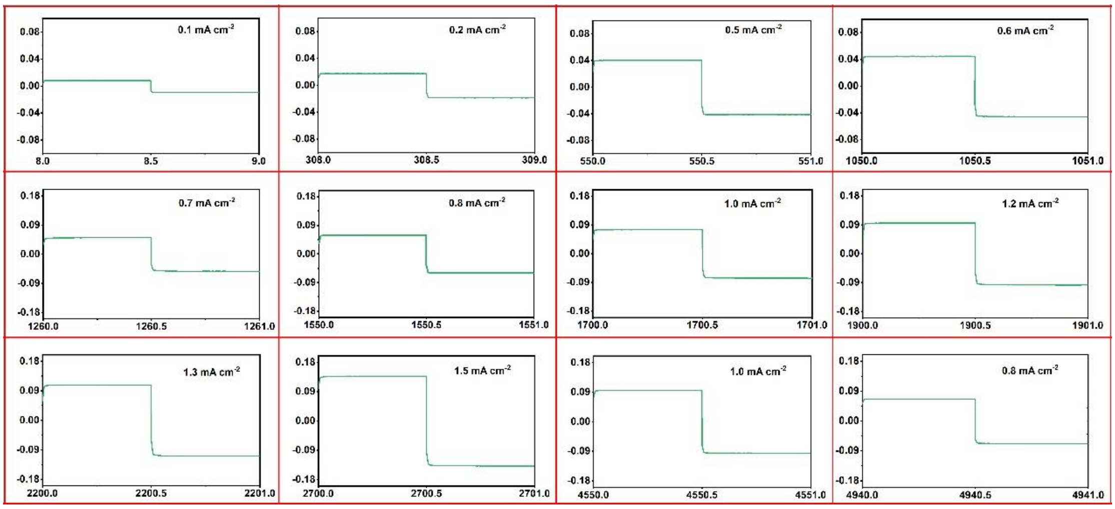

Figure S17. Polarization voltage with different current density

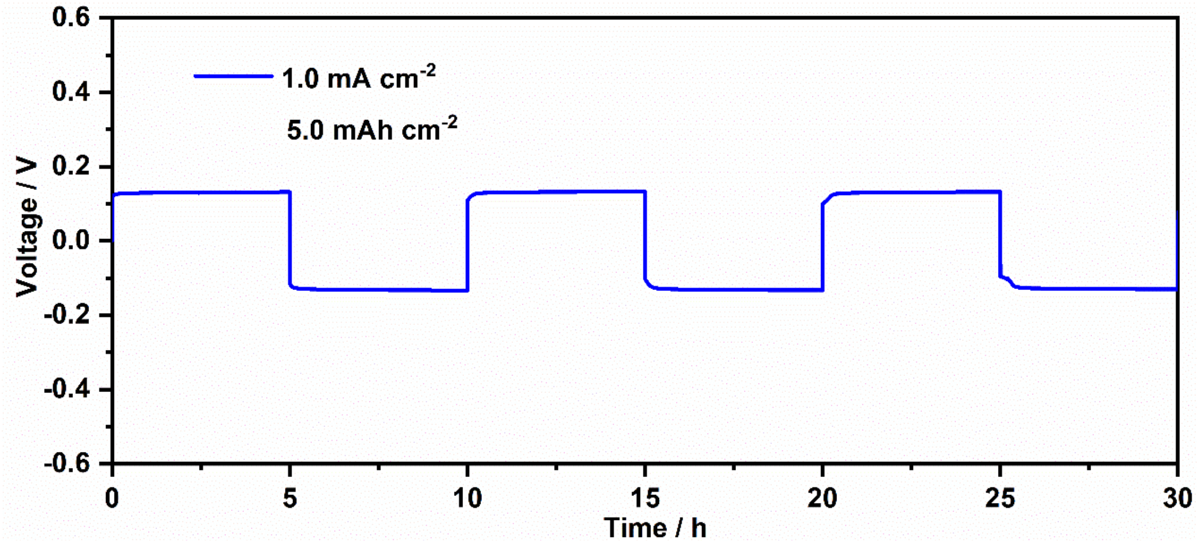

Figure S18. Galvanostatic cycling performance of the Li|BM-LLZTO|Li cell under 5 mAh cm^{-2} at RT.

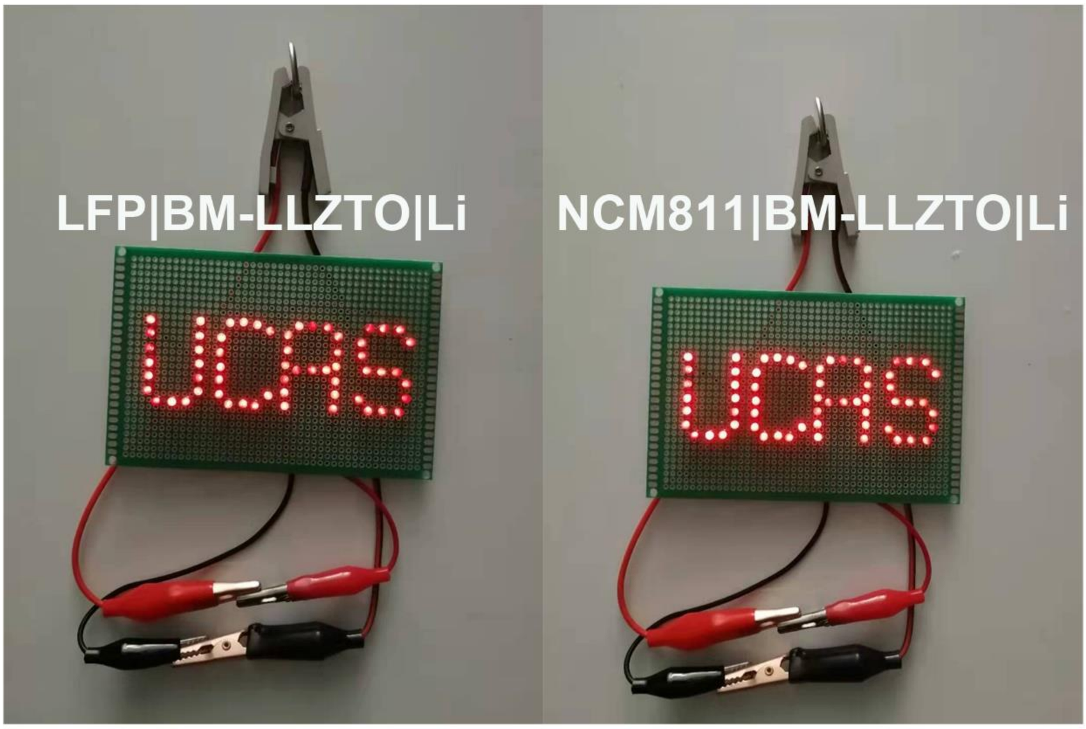

Figure S19. A structural LFP|BM-LLZTO|Li and NCM811| BM-LLZTO ||Li cell lighting a LED bulb.

Table S1 Calculation of Gibbs free energy of reaction

**LaF3+3Li→3LiF+La**			
Temperature / K	LaF_3	LiF	$\Delta_r G_m$ /KJ mol^{-1}
	$\Delta_f G_m$ / KJ mol^{-1}	$\Delta_f G_m$ / KJ mol^{-1}	
298.15	-1623.78	-588.661	-142.205
300	-1623.31	-588.486	-142.15
400	-1598.15	-579.036	-138.963
500	-1573.38	-569.333	-134.621
600	-1548.91	-559.319	-129.043

1. The Gibbs free energy is calculated according to the following formula

$\mathbf{\Delta_r G_m = \sum_i \nu i \, \Delta_f G_m (resultant) - \sum_i \nu i \, \Delta_f G_m (reactant)}$

2. Calculation of Gibbs free energy of chemical reaction equation:

   $Li_2CO_3+2HF \rightarrow 2LiF+H_2O+CO_2$

$\Delta_r G_m^\theta = 2\Delta_f G_m^\theta(LiF , s)+\Delta_f G_m^\theta(H_2O , l)+\Delta_f G_m^\theta(CO_2 , g)-$

$\Delta_f G_m^\theta(Li_2CO_3 , s)-2\Delta_f G_m^\theta(HF , g)$

$=2\times(-588.661)+(-237.141)+(-394.364)-(-1132.123)-2\times(-274.645)$

$=-127.414$ KJ mol^{-1}

3. The data in the table are from *Thermochemical Data Of Pure Substances*

- **The calculated Gibbs free energy with x=3.25, y=1.5**

$Li_{6.5}La_3Zr_{1.5}Ta_{0.5}O_{12}+9.5HF \rightarrow Li_{3.25}La_{1.5}Zr_{1.5}Ta_{0.5}O_{7.25}F_{1.75}+3.25LiF+1.5LaF_3$
$+4.75H_2O$

$Li_{52}La_{24}Zr_{12}Ta_4O_{96} +76HF \rightarrow Li_{26}La_{12}Zr_{12}Ta_4O_{58}F_{14} + 26LiF +12LaF_3+38H_2O$

Table S2 The calculated energies (eV) for x=3.25, y=1.5 reaction

	$G(Li_{26}La_{12}Zr_{12}Ta_4O_{58}F_{14})$	G(LiF)	$G(LaF_3)$	$G(H_2O)$
Product	-934.80681414	-9.861900675	-27.41518001	-14.22430984
	$G(Li_{52}La_{24}Zr_{12}Ta_4O_{96})$	G(HF)		
Reactant	-1375.32127319	-8.507914815		

$\Delta G=[G(Li_{26}La_{12}Zr_{12}Ta_4O_{58}F_{14})+26G(LiF)+12G(LaF_3)+38G(H_2O)-G(Li_{52}La_{24}Zr_{12}Ta_4O_{96})-76G(HF)]/188$

$=\{[-935.14426512+26\times(-9.861900675)+12\times(-27.41518001)+38\times(-14.22430984)]-[(-1375.32127319)+76\times(-8.507914815)]\}/188$

= -0.208174562 eV/atom

= -20.01678477 KJ/mol

- **The calculated Gibbs free energy with x=6.5, y=3**

$Li_{6.5}La_3Zr_{1.5}Ta_{0.5}O_{12}$ + 19HF → $Zr_{1.5}Ta_{0.5}O_{2.5}F_{3.5}$ + 6.5LiF +3LaF_3+9.5H_2O

$Li_{52}La_{24}Zr_{12}Ta_4O_{96}$ + 152HF →$Zr_{12}Ta_4O_{20}F_{28}$ + 52LiF +24LaF_3+76H_2O

Table S3 The calculated energies (eV) for x=3.25, y=1.5 reaction

	G($Zr_{12}Ta_4O_{20}F_{28}$)	G(LiF)	G(LaF_3)	G(H_2O)
Product	-506.52347275	-9.861900675	-27.41518001	-14.22430984
	G($Li_{52}La_{24}Zr_{12}Ta_4O_{96}$)	G(HF)		
Reactant	-1375.32127319	-8.507914815		

ΔG=[G($Zr_{12}Ta_4O_{20}F_{28}$)+52G(LiF)+24G(LaF_3)+76G(H_2O)-G($Li_{52}La_{24}Zr_{12}Ta_4O_{96}$)-152G(HF)]/188

={[-506.52347275+52×(-9.861900675)+24×(-27.41518001)+76×(-14.22430984)]-[(-1375.32127319)+152×(-8.507914815)]}/188

= -0.477818356 eV/atom

= -45.94407266 KJ/mol

Table S4 Comparison of critical current densities (CCD) and cycling stabilities between our work and recent publications

Interfacial modification	Interfacial resistance (Ω cm^{-1})	CCD (mA cm^{-2})	Stability (RT) (mA cm^{-2} /lifetime in h)	Ref
Sputtering Au	101.6/167.8	0.5	0.08/150	[9]
ALD Al_2O_3	34	N/A	0.2/90	[10]
PECVD Si	127	0.2	0.1/225	[11]
ALD Al	75	0.2	0.2/42	[12]
Drawing Soft graphite	105	N/A	0.3/1000	[13]
Co-sputtering Cu_6Sn_5	236	N/A	0.25/300	[14]
Polishing MoS_2	14@100℃	2.2@100℃	0.8/240@100℃	[15]
PECVD Li_3N	175	N/A	0.1/210	[16]
Sputtering Cu_3N	83.4	1.2	0.5/400	[17]
HCl treatment	26	N/A	0.2/700	[18]
NH_4F treatment	45	1.4	0.5/200	[19]
liquid metal	19.5	1.7	0.2/10000@60℃	[20]
BNNS treatment	9	1.5	0.3/380	[21]
HCl-LiF treatment	11.6	1.8	0.5/1000	[22]
3D-micropatterned	39.5	0.7	0.5/500	[23]
$Li_2PO_2F_2$	5.1	1.2	0.6/1500	[24]
$NH_4H_2PO_4$	13	1.2	0.1/1000	[25]
**HF treatment**	**3**	**2.7**	**0.1~1.5/12000**	**This work**

Table S5 Comparison of battery performance with electrolyte infiltrating cathode electrolyte interface

ref	Cathode composition	current	Voltage range	First cycle discharge capacity	Number of cycles	Capacity retention
[26]	LFP	1C	2.5~4	136	50	96.2%
[27]	LFP	0.5C	2.5~4	158.9	100	N/A
[28]	LCO	0.4C	2.8~4.2	135.1	70	64.7%
[21]	LFP	0.5C	2.5~3.8	150	100	90%
[29]	LFP	0.5C	2.5~4	150	100	90%
[30]	LFP	0.05 mAcm^{-2}	2.8~4	N/A	N/A	N/A
[19]	LFP	170 μAcm^{-2}	2.5~4	N/A	50	N/A
	NCM622	100 μAcm^{-2}	2.8~4.3	N/A	30	N/A
**This work**	**LFP**	**1C**	**3.0~4.2**	**146**	**900**	**85.4%**
	**NCM811**	**0.5C**	**3.0~4.3**	**181**	**200**	**89%**

References

[1] G.Kresses, J. Furthmuller, *Comput.Mater.Sci* **1996**,6,15.

[2] J.Perdew, K. Burke, M. Ernzerhof, *Phys. Rev. Lett* **1996**,77, 3865.

[3] P.Blochl, E. Blochl, PE. Blochl, *Matter Mater. Phys* **1994**,50, 17953.

[4] G.Kresses, D. Joubert, *Phys. Rev. B: Condens. Matter Mater. Phys* **1999**,59,1758.

[5] G.Henkelman, H. Jonsson, *J. Chem. Phys* **2000**,113, 9901.

[6] G.Henkelman, H. Jonsson, *J. Chem. Phys* **2000**,113, 9978.

[7] L.Chen, et al. *ACS Appl. Mater. Interfaces* **2018**,10, 26972.

[8] Z.Lv, C. Cheng, Y. Cheng, X. Chen, G. Ji, *Computational Materials Science* **2014,**89. 57.

[9] C-L.Tsai, V. Roddatis, C.V. Chandran, Q. Ma, S. Uhlenbruck, M. Bram, P. Heitjans, O.Guillon, *ACS Appl. Mater. Interfaces* **2016**,8,10617.

[10] X.Han, Y. Gong, K. Fu, X. He, G-T. Hitz, J. Dai, A. Pearse, B. Liu, H. Wang, G.Rubloff, Y. Mo, V. Thangadurai, E. Wachsman, L. Hu, *Nature Materials* **2017,**16,572.

[11] W.Luo, Y. Gong, Y. Zhu, K. Fu, J. Dai, S. Lacey, C. Wang, B. Liu, X. Han, Y. Mo, E.Wachsman, L.Hu, *J. Am. Chem. Soc.* **2016,**138, 12258.

[12] K.Fu, Y. Gong, B. Liu, Y. Zhu, S. Xu, Y. Yao, W. Luo, C. Wang, D. Lacey, J. Dai,Y.Chen, Y. Mo, E. Wachsman, L. Hu, *Sci. Adv* **2017,3**, e1601659.

[13] Y.Shao, H. Wang, Z. Gong, D. Wang, B. Zheng, J. Zhu, Y. Liu, Y-S. Hu, X. Guo, H.Li, X. Hua, Y. Yang, C-W. Nan, L. Chen, *ACS Energy Lett* **2018**,3, 1212.

[14] W. Feng, X. Dong, Z. Lai, X. Zhang, Y. Wang, C. Wang, J. Luo, Y. Xia, *ACS Energy Lett* **2019**,4, 1725.

[15] J. Fu, P. Yu, N. Zhang, G. Ren, S. Zheng, W. Huang, X. Long, H. Li, X. Liu, *Energy Environ. Sci* **2019**,12, 1404.

[16] H.Xu, Y. Li, A. Zhou, N. Wu, S. Xin, Z. Li, JB. Goodenough, *Nano Lett* **2018**,18, 7414.

[17] H.Huo, Y. Chen, R. Li, N. Zhao, J. Luo, J. Silva, R. Mucke, P. Kaghazchi, X. Guo, X. Sun, *Energy Environ. Sci* **2020**,13, 127.

[18] H. Huo, Y. Chen, N. Zhao, X. Lin, J. Luo, X. Yang, Y. Liu, X. Guo, X. Sun, *Nano Energy* **2019**,61, 119.

[19] H.Duan, W. Chen, M. Fan, W. Wang, Y. Lu, S. Tan, X. Chen, Q. Zhang, S. Xin, L.Wan, Y. Guo, *Angew. Chem. Int. Ed* **2020**,59, 12069.

[20] J.Meng, Y. Zhang, X. Zhou, M. Lei, C. Li, Li2CO3-affiliative mechanism for air-accessible interface engineering of garnet electrolyte via facile liquid metal painting. *Nat. Communications* **2020**,11, 3716.

[21] J.Wen, Y. Huang, J. Duan, Y. Wu, W. Luo, L. Zhou, C. Hu, L. Huang, X.Zheng, W.Yang, Z. Wen, Y. Huang, *ACS Nano* **2019**,13. 12.

[22] Y.Ruan, Y. Lu, Y. Li, C. Zheng, J. Su, J. Jin, T. Xiu, Z. Song, M. Badding, Z.Wen, *Adv. Funct. Mater* **2021**,31, 5.

[23] R.Xu, F. Liu, Y. Ye, H.Chen, R. Yang, Y. Ma, W. Huang, J. Wan, Y. Cui, *Adv. Mater.* **2021**,33,2104009.

[24] X. Yang, S. Tang, C. Zheng, F. Ren, Y. Huang, X. Fei, W. Yang, S. Pan, Z, Gong. Y. Yong, *Adv. Funct. Mater* **2022**, 2209120.

[25] Z. Bi, Q. Sun, M. Jia, M. Zou, N. Zhao, X. Guo. *Adv. Funct. Mater* **2022**, 202208751.

[26] X.He, F. Yan, M. Gao, Y. Shi, G. Ge, B. Shen, J. Zhai, *Mater. Interfaces* **2021**,13, 42212.

[27] Y.Lu, X. Huang, Y. Ruan, Q. Wang, R. Kun, J. Yang, Z. Wen, *J. Mater. Chem. A* **2018**,6, 18853.

[28] J.Zhang, R. Yu, J. Li, H. Zhai, G. Tan, X. Tang, *Energy Environ. Mater* **2021**,0, 1.

[29] J.Duan, W.Wu, A. Nolan, T. Wang, J. Wen, C. Hu, Y. Mo, W. Luo, Y. Huang, *Adv. Mater* **2019**,31, 1807243.

[30] X.Fu, T. Wang, W. Shen, M. Jiang, Y. Wang, Q. Dai, D. Wang, Z. Qiu, Y. Zhang, K.Deng, K. Zeng, Q. Zeng, N. Zhao, X. Guo, Z. Liu, J. Liu, Z. Peng, *Adv. Mater* **2020**,32, 26.
